# SARS-CoV-2 Quasispecies Provides an Advantage Mutation Pool for the Epidemic Variants

**DOI:** 10.1128/spectrum.00261-21

**Published:** 2021-08-04

**Authors:** Fengming Sun, Xiuhua Wang, Shun Tan, Yunjie Dan, Yanqiu Lu, Juan Zhang, Junli Xu, Zhaoxia Tan, Xiaomei Xiang, Yi Zhou, Weiwei He, Xing Wan, Wei Zhang, Yaokai Chen, Wenting Tan, Guohong Deng

**Affiliations:** a Department of Infectious Diseases, Southwest Hospital, Third Military Medical University (Army Medial University), Chongqing, China; b Division of Infectious Diseases, Chongqing Public Health Medical Center, Chongqing, China; c Beijing Novogene Company Limited, Beijing, China; d Chongqing Key Laboratory for Research of Infectious Diseases, Chongqing, China; Fundacio irsiCaixa

**Keywords:** quasispecies, SARS-CoV-2, COVID-19, spike gene

## Abstract

The dynamics of quasispecies afford RNA viruses a great fitness on cell tropism and host range. To study the quasispecies features and the intra-host evolution of SARS-CoV-2, we collected nine confirmed patients and sequenced the haplotypes of spike gene using a single-molecule real-time platform. Fourteen samples were extracted from sputum, nasopharyngeal swabs, or stool, which in total produced 283,655 high-quality circular consensus sequences. We observed a stable quasispecies structure that one master mutant (mean abundance ∼0.70), followed by numerous minor mutants (mean abundance ∼1.21 × 10^−3^). Under high selective pressure, minor mutants may obtain a fitness advantage and become the master ones. The later predominant substitution D614G existed in the minor mutants of more than one early patient. An epidemic variant had a possibility to be independently originated from multiple hosts. The mutant spectrums covered ∼85% amino acid variations of public genomes (GISAID; frequency ≥ 0.1) and likely provided an advantage mutation pool for the current/future epidemic variants. Notably, 32 of 35 collected antibody escape substitutions were preexistent in the early quasispecies. Virus populations in different tissues/organs revealed potentially independent replications. The quasispecies complexity of sputum samples was significantly lower than that of nasopharyngeal swabs (*P* = 0.02). Evolution analysis revealed that three continuous S2 domains (HR1, CH, and CD) had undergone a positive selection. Cell fusion-related domains may play a crucial role in adapting to the intrahost immune system. Our findings suggested that future epidemiologic investigations and clinical interventions should consider the quasispecies information that has missed by routine single consensus genome.

**IMPORTANCE** RNA virus population in a host does not consist of a consensus single haplotype but rather an ensemble of related sequences termed quasispecies. The dynamics of quasispecies afford SARS-CoV-2 a great ability on genetic fitness during intrahost evolution. The process is likely achieved by changing the genetic characteristics of key functional genes, such as the spike glycoprotein. Previous studies have applied the next-generation sequencing (NGS) technology to evaluate the quasispecies of SARS-CoV-2, and results indicated a low genetic diversity of the spike gene. However, the NGS platform cannot directly obtain the full haplotypes without assembling, and it is also difficult to predict the extremely low-frequency variations. Therefore, we introduced a single-molecule real-time technology to directly obtain the haplotypes of the RNA population and further study the quasispecies features and intrahost evolution of the spike gene.

## INTRODUCTION

Coronaviruses are enveloped, single, and positive-stranded RNA viruses that can be further classified into four genera: *Alphacoronavirus*, *Betacoronavirus*, *Gammacoronavirus*, and *Deltacoronavirus* (1). Betacoronaviruses have a zoonotic potential and can be transmitted from animals to humans causing a novel, severe respiratory disease (2). The severe acute respiratory syndrome (SARS) caused by SARS-related coronavirus (SARS-CoV) broke out in 2002 and resulted in nearly 8,000 laboratory-confirmed cases and 800 deaths globally (3, 4). The natural reservoir of SARS-CoV was presumed to be *Rhinolophus sinensis* (coronavirus strains RsSHC014 and Rs3367) (5). In 2012, another betacoronavirus caused the outbreak of Middle East respiratory syndrome (MERS-CoV) (6, 7). By November 2019, MERS had resulted in 2,494 cases and 858 deaths worldwide (8). The origin of MERS-CoV remains elusive, but studies have shown that humans were infected by direct or indirect contact with the infected dromedary camels in Arabian Peninsula (WHO). Although the two large-scale epidemics have gradually subsided, the threat from zoonotic *betacoronaviruses* always exists due to its inherent characteristics (genetic diversity) and frequently contact between humans and animals (hunting and domestication).

In December 2019 in Wuhan, China, cases of an unknown pneumonia emerged with the main symptoms of fever, fatigue, cough, and difficult breathing (COVID-19). The epidemic was confirmed to be caused by a novel betacoronavirus called SARS-CoV-2 (9). Genomic sequences of SARS-CoV-2 showed a high similarity (∼0.96) to bat coronavirus RaTG13, indicating a potential bat origin of the virus (10, 11). COVID-19 is still a pandemic; according to the World Health Organization (WHO), there were over 110 million confirmed cases and 2.5 million deaths worldwide as of March 2021. Although antiviral drugs and vaccines are under development and some of them have been used clinically (12–15), the actual work will face many challenges, such as long clinical trials and the risk of poor effects caused by genetic instability (16). For long-term effective prevention of the disease, it is crucial to study and understand the underlying genetic dynamics of SARS-CoV-2.

At the consensus genome level, SARS-CoV-2 has been reported with limited variations (17–20). The RNA virus population in a host does not consist of a consensus single haplotype, but rather an ensemble of related sequences termed quasispecies. The dynamic changes of quasispecies affords RNA viruses a greater probability of changing their cell tropism or host range or to overcome internal or external selective constraints (21). The process is likely achieved by changing the genetic characteristics of key functional genes, such as the spike glycoprotein (22). The spike protein has been recognized as a potential therapeutic and vaccine target. Previous studies indicated that healed patients revealed high titers of SARS-CoV-2 neutralizing antibodies, and antibodies against the spike protein likely provided a main contribution to the protective effect (16, 23). The spike mutation of D614G is widely spread in infected patients, and its infection ability is 2.6 to 9.3 times stronger than the early ancestral haplotypes (24–26). Another currently pandemic variant VOC 202012/01 (lineage B.1.1.7) defined by 17 mutations (eight of which are in the spike protein) has a 43 to 90% higher reproduction number than preexisting variants (27), and the mutations of spike protein are highly associated with a high mortality rate (28). Spike gene could be primarily determined the strong infection capability of SARS-CoV-2 through binding the receptor angiotensin-converting enzyme 2 (ACE2) of host cells (29, 30). By studying spike gene at the quasispecies level, researchers can not only observe the intrahost evolution of SARS-CoV-2 but also widely predict the potential fitness mutations. Several studies have applied next-generation sequencing (NGS) technology to evaluate these quasispecies features, and the data revealed a low genetic diversity of the spike gene (16 to 87 single-nucleotide variations [SNVs] or deletions) (31–34). However, the NGS platform cannot directly obtain full haplotypes without assembling, and it is also difficult to predict the extremely low-frequency variations because the high depth of coverage required to differentiate between sequencing errors and true polymorphisms (35).

Therefore, the aim of the present study is to investigate the SARS-CoV-2 spike gene quasispecies using PicBio single-molecule real-time (SMRT) technology. Two single-source infection sets, including nine first- to third-generation patients, were collected, and then 14 RNA samples were extracted from nasopharyngeal swabs (NS), sputum (SP), and stool (ST). A circular consensus sequence (CCS)-related algorithm was applied to obtain high-quality quasispecies haplotypes of the spike gene. Based on this, the main quasispecies features (structure, complexity, and genetic distance) were investigated and further compared among different samples. The selective pressures and potential fitness variations of the spike gene were also evaluated. Our results provide insights into the intrahost evolution of SARS-CoV-2 and also provide important information for its future prevention.

## RESULTS

### Ultradeep sequencing of the SARS-CoV-2 spike gene.

**FIG 1:**
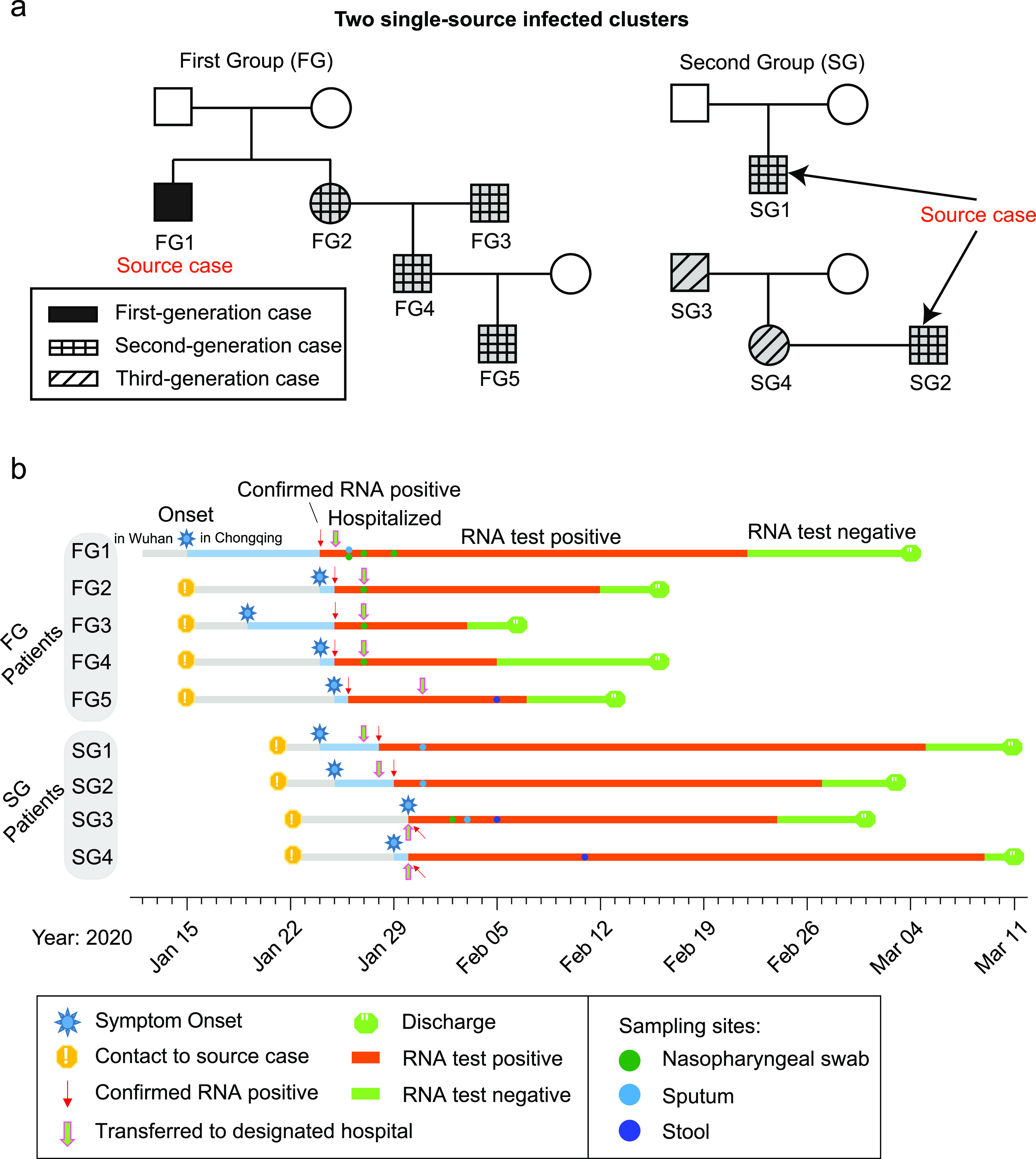
Details of participants and the samples collected in this study. (a) Family trees of patients with COVID-19. A total of nine patients were assigned to two different single-source infection groups. Each group contain one main family cluster. (b) Timeline of exposure history to index patients and medical course of the participants.

**FIG 2:**
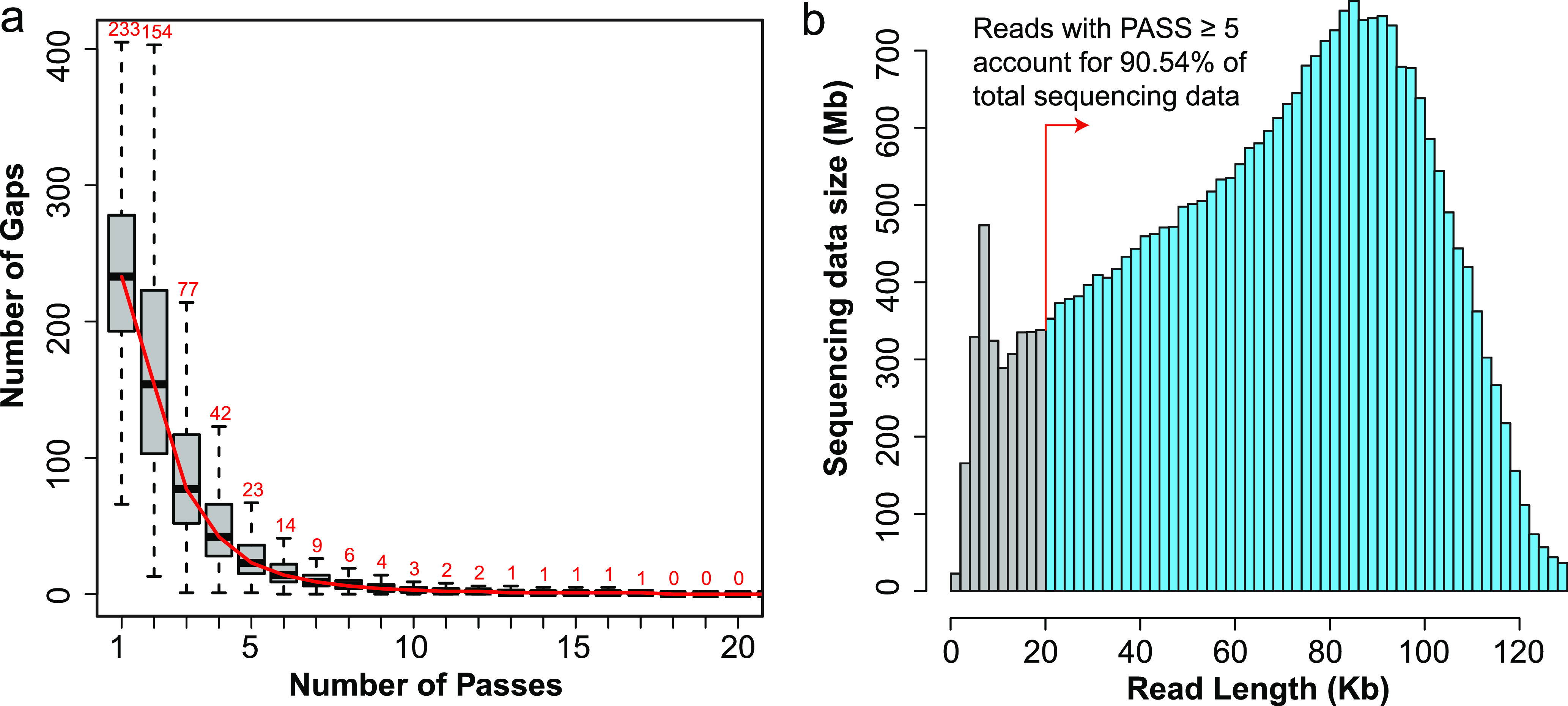
Quality assessments of CCSs. (a) Number of gaps in CCSs under different circular sequencing passes. When pass is equal to 5, the number of gaps in CCS decreased to a stable low value, which is close to completing self-correction. (b) Meanwhile, accumulated length of subreads with CCS pass ≥ 5 accounted for 90.54% of total sequencing data.

A total number of 14 samples were collected form two spread clusters (or groups), the first group (FG) included five first-to-third infected patients and the second group (SG) included 4 s-to-third infected patients (Fig. 1; see also Table S1 in the supplemental material). The SMRT platform produced 26.97-Gb raw sequencing reads (mean ± the standard deviation, 1.93 ± 0.88 Gb) that resulted in 379,079 CCSs (5,044 to 52,739) (see Table S2). Metagenomic sequencing based on NGS was used to evaluate the effectiveness of amplification primers. Reads mapping results showed that more than 98% of the SARS-CoV-2 genomes had no mutations in the primer regions (see Fig. S1). With the increase of pass number, the main sequencing errors (gaps, i.e., insertions and deletions) could be self-modified by generating the CCSs. The statistical results showed that the average gap number of CCS was reduced to a stable low level (median, 23 [accounting for ∼0.60% of the full length]) when the pass number is equal to 5 (Fig. 2a). Therefore, only CCSs with a pass number of ≥5 were considered for further analysis. Below this threshold, we obtained 283,655 high-quality CCSs (74.83%) that covered ∼90.54% of the total sequencing data (Fig. 2b; see also Table S2).

### Observation of a stable quasispecies structure and a potential advantage mutation.

There were a total of 282,866 (99.72%) qualified CCSs contained the full length of spike gene. According to sequence similarity, these CCSs could merge into 25,490 nonredundant sequences (defined as mutants) (Table 1). Compared to the reference of an early ancestral strain (Wuhan-Hu-1, 5 January 2020; GenBank accession no. MN908947.3), all mutants accumulated 8,976 SNVs and 6,458 amino acid changes (see Table S3). All samples revealed a similar quasispecies structure of one predominant master mutant (abundance, 0.57 to 0.77; mean, 0.70 ± 0.06) combined with numerous minor mutants (abundance, 0.05 to 2.55 × 10^−3^; mean, 1.21 ± 7.33 × 10^−3^) (see Fig. S2). Two single-source infection groups contained different master mutants that mainly revealed one nucleotide difference at nucleotide (nt) 3118, where the master mutants of FG (MFG) and SG (MSG) harbored nucleotides T and G, respectively (Table 1).

**TABLE 1:** Information of SARS-CoV-2 quasispecies

Sample^*a*^	No. of CCSs^*b*^	All mutants^*c*^						Master mutant (most abundant)^*e*^		
		No. of mutants	No. of accumulated SNVs	Nonsense SNVs (%)	Missense SNVs (%)	Mean distance^*d*^	Sn	Included CCS	Abundance^*f*^	3118[T/G] genotype^*g*^
SG1-0131-SP	32,020	3,806	3,236	4.08	76.70	8.00E–04	0.43	23,166	0.72	G(V^1040^)
SG2-0131-SP	28,872	4,776	4,717	4.30	74.54	7.99E–04	0.38	21,483	0.74	G(V^1040^)
SG4-0211-ST	10,558	1,681	1,742	4.59	78.76	9.37E–04	0.51	5,975	0.57	G(V^1040^)
SG3-0203-NS	12,073	1,939	1,998	3.95	78.28	9.66E–04	0.50	7,482	0.62	G(V^1040^)
SG3-0205-ST	27,952	3,244	2,765	4.27	77.14	9.12E–04	0.49	18,843	0.67	G(V^1040^)
SG3-0206-SP	39,123	4,675	4,010	4.24	74.46	8.26E–04	0.40	28,978	0.74	G(V^1040^)
FG1-0126-NS	12,346	1,171	1,177	2.80	75.79	7.68E–04	0.49	9,361	0.76	T(F^1040^)
FG1-0126-SP	26,378	3,675	3,373	3.82	75.93	9.24E–04	0.43	17,107	0.65	T(F^1040^)
FG1-0127-NS	12,912	1,298	1,267	3.63	76.64	8.20E–04	0.48	9,417	0.73	T(F^1040^)
FG1-0129-NS	8,852	1,314	1,415	3.53	75.90	6.70E–04	0.37	6,838	0.77	T(F^1040^)
FG2-0127-NS	30,525	2,596	2,434	3.86	75.76	8.17E–04	0.47	22,905	0.75	T(F^1040^)
FG3-0127-NS	18,865	1,949	1,914	3.24	74.97	8.11E–04	0.48	13,204	0.70	T(F^1040^)
FG4-0127-NS	18,722	1,929	1,871	3.15	75.36	7.57E–04	0.44	13,917	0.74	T(F^1040^)
FG5-0205-ST	3,668	367	439	5.01	78.82	9.01E–04	0.64	2,493	0.68	T(F^1040^)

a Sample names were constructed using the cluster group (SG or FG) and individual number, the 2020 date for the sample, and the sample type (SP, means sputum; ST, stool; and NS, nasopharyngeal swab).

b CCSs that containing the full length of the Spike gene.

c A set of CCSs with the same haplotype were merged into one kind of mutant.

d Mean distance between each pair of mutants (substitutions per site).

e The master mutant was defined as a mutant containing the largest number of CCSs.

f Abundances were calculated by counting the ratio of mutant related CCSs.

g The genotype at nt 3118 and the corresponding nucleotide and amino acid (in parentheses) are listed.

Relative to the reference of Wuhan-Hu-1, MFG contained a SNV of 3118G→T, but MSG remained unchanged at the same site. Compared to the published GenBank genome sequences (as of March 2021), the 3118G→T variation never emerged, but it did emerge in some strains from North America after October 2020 (∼10 months after the sampling date of this study) (see Fig. S3 and Table S4 in the supplemental material). The number of such variants is still rising. However, the origins of the 3118G→T was different in that MFG had likely evolved from the Wuhan-Hu-1 strain, but the variants from North America had evolved from strains with the dominant SNV of 1841A→G (D614G). Our samples were taken around February 2020, which likely suggested that the spread chain of MFG had been terminated at an early stage. The 3118G→T variation could result in a valine-to-phenylalanine amino acid change in the S2 domain. By simulating a three-dimensional protein model, we were able to observe that the mutation could further change its corresponding R group (see Fig. S4 and S5).

### The mutant spectra and conversion event between master and minor mutants.

**FIG 3:**
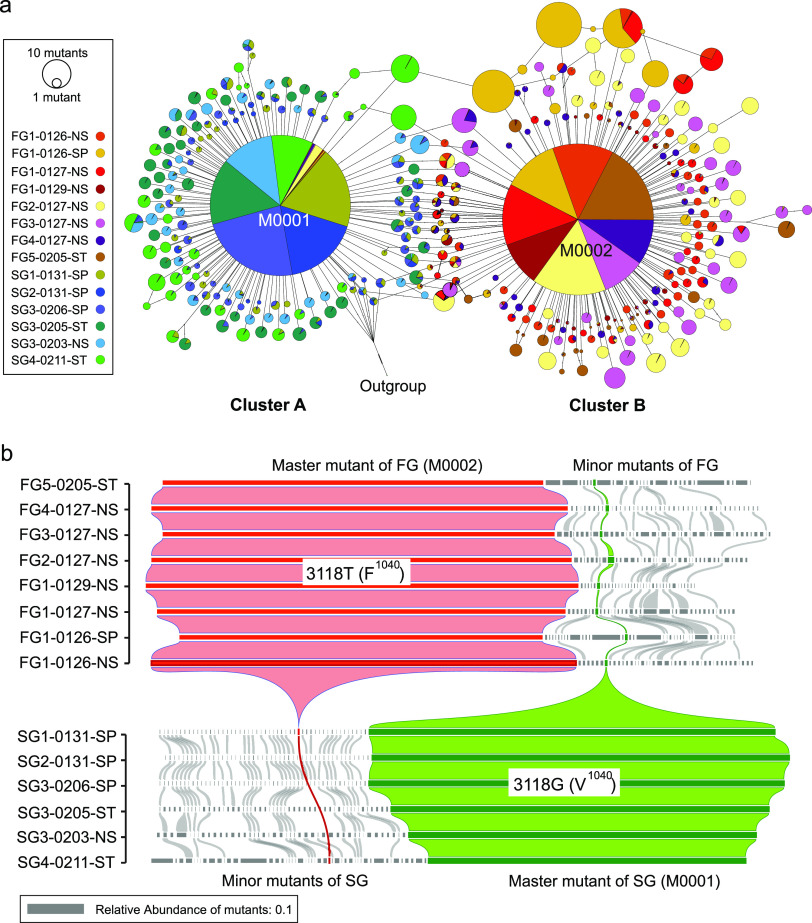
Haplotype clusters and abundance distribution of the quasispecies mutants. (a) All of the top 30 abundant mutants of each sample were used to perform haplotype clustering analysis. Samples were distinguished by different colors, where FG samples are marked with warm colors and SG samples are marked with cold colors. Two clusters are mainly assigned to FG (group B) and SG (group A), respectively. (b) Mutants that exist in any samples with the top 30 abundance are listed, and the abundance value is indicated by the width of the rectangle. Mutants that are shared by adjacent samples are connected with a Bezier curve (gray), whereas two master mutants are highlighted in red and green. A conversion phenomenon is observed between the minor and master mutants.

Haplotype clustering was performed using the top 30 abundant mutants of each sample, and mutant spectra centered by the master one were observed (see Fig. S2). Assessment of the topological structure and haplotype alignments revealed that most minor mutants of each sample (96.67 to 100%) were likely evolved from the master (see Fig. S2). All 14 mutant spectra could have further merged into two haplotype clusters (clusters A and B) (Fig. 3a). Due to the variation 3118G→T, cluster A and B mutants were mainly from SG and FG, respectively (see Fig. S6). Within the same single-source infection group, a high number of 234 (∼67%) mutants could be shared by at least two samples (FG, 112; SG, 122) (see Table S5), while only 27 minor mutants (7.71%) could stably exist in more than 80% samples (FG, 7; SG, 22) (see Table S5). Notably, a conversion event was observed that the master mutant of one group (M0001 or M0002) could exist as minor mutants in the other group (Fig. 3).

### The mutant spectra were likely the advantage variation pool of the epidemic strains.

**FIG 4:**
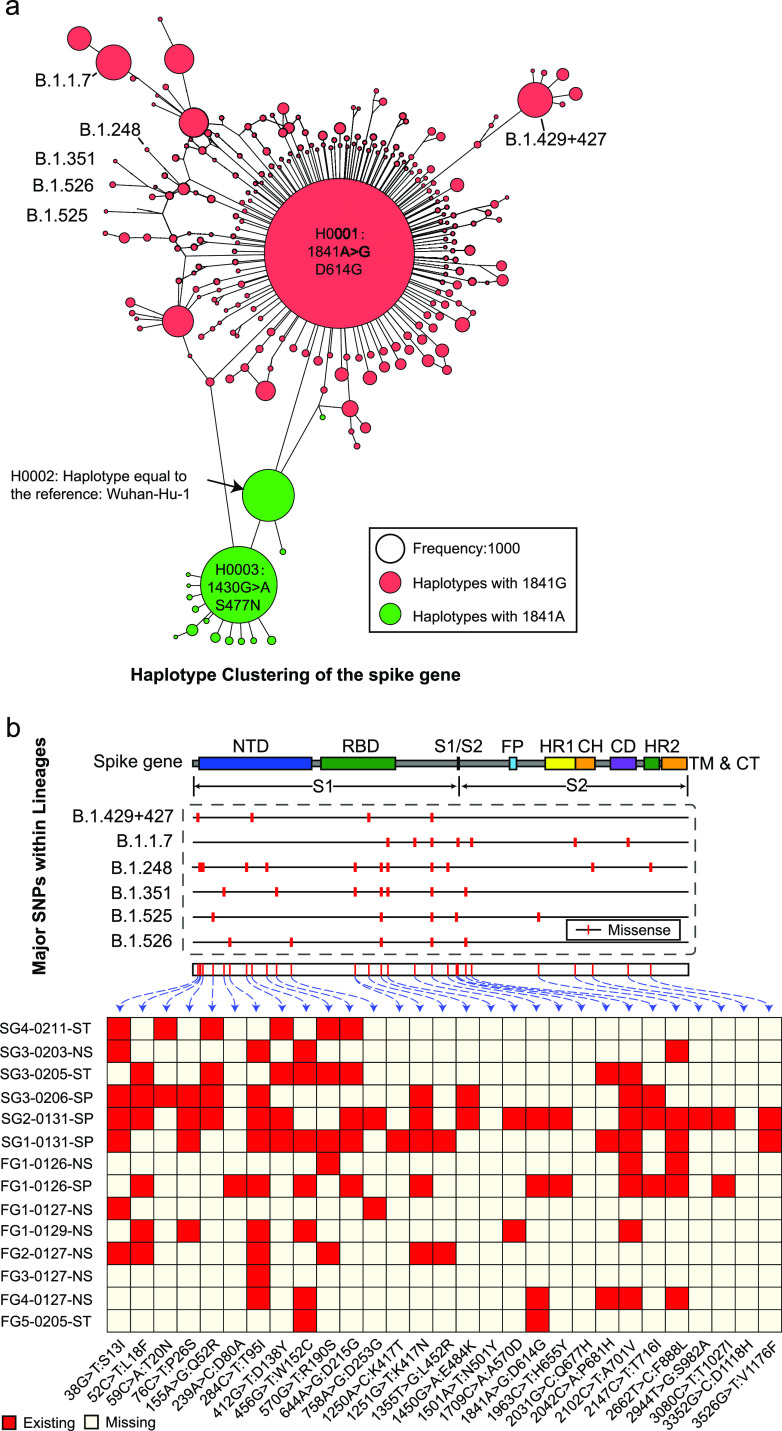
Comparisons for SNVs between current epidemic variants and the quasispecies mutants. (a) Haplotype clustering of the public consensus genomes of GenBank (as of 1 April 2020; occurrence ≥ 10). Three main clusters are found and centered by three haplotypes H0001-H0003. Six current epidemic variant lineages are highlighted and evolved from a predominant variant with D614G mutations. (b) A total of 29 advantageous variations were found in the six lineages, 90% of which were found in the quasispecies mutants of early-infected patients. Each functional domain is marked by a rectangle. NTD, N-terminal domain; RBD, receptor-binding domain; S1/S2, protease cleavage site; FP, fusion peptide; HR1, heptad repeat 1; CH, central helix; CD, connector domain; HR2, heptad repeat 2; TM, transmembrane domain; CT, cytoplasmic tail.

The conversion phenomenon between the minor and master mutants was also observed based on 68,769 public SARS-CoV-2 genomes (GenBank, 1 April 2021). The consensus genome sequence reflects the high-abundance master mutant in quasispecies. Three hub haplotypes (H0001 to H0003) were found in the clusters (Fig. 4a), where all could exist as the minor mutants in the SARS-CoV-2 quasispecies (see Fig. S7 and Table S6 in the supplemental material). Strains of H0001 (with D614G) disseminated rapidly and further evolved into several epidemic variants, such as B.1.1.7, B.1.248, B.1.351, B.1.526, B.1.525, and B.1.429+427. Based on the GISAID database (2 April 2021), the six variants contained 29 major SNVs (frequency ≥ 0.60), where 26 (∼90%) had already existed in the quasispecies of early-infected patients (Fig. 4b; see also Table S7). In particular, the predominant amino acid variation, D614G, was found in 19 minor mutants from SG2-0131-SP, FG1-0126-SP, FG4-0127-NS, and FG5-0205-ST (see Fig. S8). We also found that ∼91% of 35 potential substitutions relating to the antibody escape have already existed in one or more early-infected patients (see Table S8). Further analysis showed that more than 85% of 1,987 accumulated amino acid variations of the spike gene (GISAID database, 2 April 2021; frequency ≥ 0.1) could also exist in the mutant spectrums (see Table S9). The mutant spectra could provide the advantage SNV pool for the current or future prevalent variants. SNVs with high abundance or frequency likely had a high fitness advantage and should be given more attention. Finally, we obtained 562 missense SNVs that existed in the top 30 abundant mutants or occurred more than 15 times (see Tables S10 and S11 in the supplemental material).

### Different sampling sites reveal different quasispecies features.

**FIG 5:**
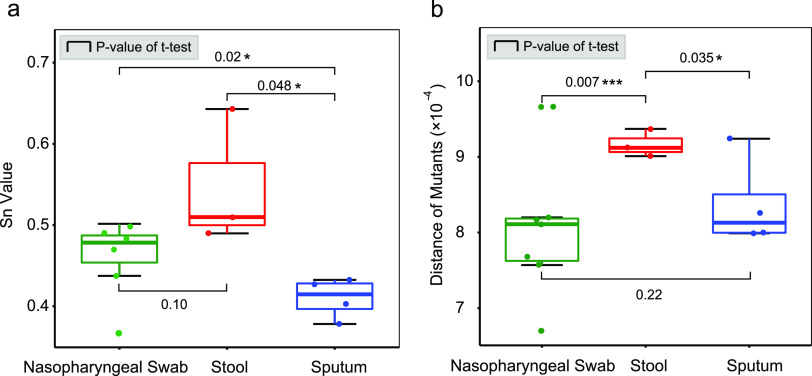
Comparisons of quasispecies features between different sampling sites. (a) Evaluations of quasispecies complexity for three sampling sites. The value of standardized Shannon entropy (Sn) was calculated, and sputum samples contain the significantly lowest Sn value compared to others. (b) Genetic distances of quasispecies mutants. Stool samples reveal significantly higher genetic distances than other two sampling sites.

Quasispecies features were determined based on the quasispecies complexity and distance of mutants. The quasispecies complexity of sputum (SP) samples was significantly less than those of other sampling sites of nasopharyngeal swabs (NS) and stool (ST) (*t* test: *P* = 0.02 and 0.048), where the mean Shannon entropy (Sn) values were 0.41 ± 0.02, 0.46 ± 0.05, and 0.55 ± 0.08 for SP, NS, and ST, respectively (Fig. 5a). For mutant distance, the values for ST samples were higher than for SP and NS samples (*t* test: *P* = 0.035 and 0.007), and the corresponding mean distances were (9.17 ± 0.18) × 10^−4^, (8.01 ± 0.90) × 10^−4^, and (8.37 ± 0.59) × 10^−4^ for ST, SP, and NS, respectively (Fig. 5b). ST samples revealed the largest value of both quasispecies complexity and mutant distance. Further analysis showed that the proportions of missense mutations (mean values: ST = 78.76%, NS = 75.90%, and SP = 75.24%) and nonsense mutations (mean values: ST = 4.59%, NS = 3.53%, and SP = 4.16%) were also higher in ST samples than in other samples (see Fig. S9).

### Evaluating the selective pressure for each function domain.

**FIG 6:**
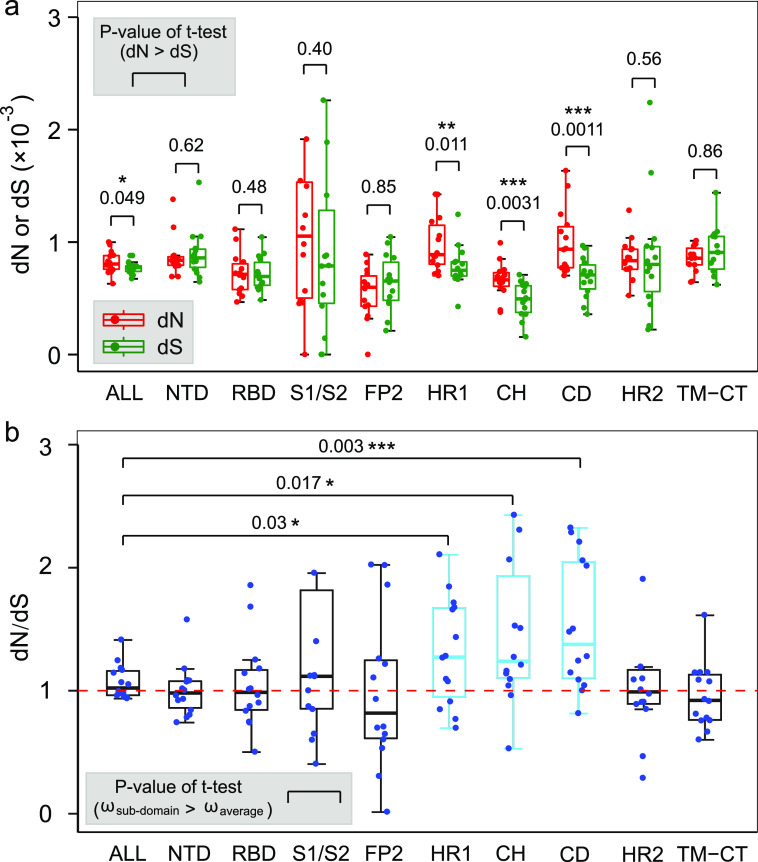
Estimation of the selective pressure for each functional domains of the spike protein. (a) Comparisons between the *dN* and *dS* values for all domains and the whole gene level (ALL). Three consecutive domains of HR1, CH, and CD show a mean *dN* value than significantly larger than that for *dS*. (b) Distribution of the ω (*dN/dS*) values. The ω_mean_ values for the HR1, CH, and CD domains are significantly larger than that of the whole gene level (ω_mean_ ≈ 1).

For each sample, the synonymous mutation rate (*dS*) and nonsynonymous mutation rate (*dN*) of the mutants were calculated to evaluate genetic fitness changes. All domains showed a relatively low mean *dS* (*dS*_mean_) value, (0.88 ± 0.26) × 10^−4^, and had no significant difference (*t* test, the cutoff of *P* = 0.05) (see Fig. S10a). The mean *dN* (*dN*_mean_) values for HR1 (heptad repeat 1) and CD (connector domain) were larger than for the whole gene level (*P* = 0.018 and 0.02, *t* test), while the *dN*_mean_ value for FP (fusion peptide) was less than for the whole gene level (*P* = 0.00038, *t* test) (see Fig. S10b). *dN* and *dS* were compared to measure the selective pressures. The whole gene sequences revealed a potentially positive selection, and the *dN*_mean_ value was slightly larger than the *dS*_mean_ value (*P* = 0.049, *t* test). The positive selection effect was mainly contributed by three continuous subdomains of HR1, CH (central helix), and CD, where the *dN*_mean_ value was significantly larger than the dS_mean_ value (*P =* 0.011, 0.0031, and 0.0011, respectively; *t* test) (Fig. 6a). Moreover, the ω_mean_ (*dN*_mean_/*dS*_mean_) values for these three domain regions were also significantly larger (*t* test) than for the whole gene level (ω_mean_ ≈ 1) (Fig. 6b). The FP domain seemed to undergo a potential purifying selection, where the value of ω_mean_ was obviously less than 1 (Fig. 6b).

## DISCUSSION

The SMRT strategy directly produced comprehensive and high-quality haplotypes for the whole spike gene of SARS-CoV-2. The quasispecies revealed a stable structure that one predominant master mutant combined with numerous minor mutants. The minor mutants had potential ability to obtain the fitness advantage and shift to the master one. The dominant mutations in the epidemic strains may already exist in the quasispecies of early-infected patients, and such mutations could originate from multiple hosts through intrahost virus mutating. The quasispecies mutant spectra would provide valuable information for tracing virus origins and predicting future advantageous mutations. Different tissues or organs brought different pressures to the survival of the quasispecies mutants. The cell membrane fusion-related subdomains were crucial for adapting to the host immune system. The present study could deepen our understanding of the underlying mechanisms on intrahost evolution of SARS-CoV-2.

High-quality haplotypes lay the foundation for the subsequent analysis and interpretation. The amplification primers were highly conserved (see Fig. S1 in the supplemental material), which ensured the mutants we obtained had a broad spectrum and could be close to reflect its true diversity. CCSs with a pass number of ≥5 were not only close to the best results of gap correction (Fig. 2) but also revealed a low substitute error of ∼0.023% (36). Under this threshold, the SMRT platform produced an average of ∼20,000 qualified CCSs for each sample. The clone (or haplotype) number was much more than that produced from traditional Sanger methods, such as the study of SARS-CoV quasispecies that obtained 28 full spike clones for 19 patients (37). Under the same SMRT platform and threshold (pass number ≥ 5), a recent study of HCV quasispecies produced an average 8,130 CCSs for each sample (36). These indicated that a sufficient and high-quality data set was obtained in the present study.

We observed a stable quasispecies structure that unique predominant master mutant (mean abundance, 0.70) combined with numerous low-abundance minor mutants (mean abundance, 1.21 × 10^−3^), which seemed like a mutants cloud with “master + dust” (see Fig. S2). For the spike gene level, the proportion of master mutant was larger than the SARS-CoV (<0.50 [based on fluorescent signal of Sanger sequencing]) (37) and less than the MERS-CoV (∼0.88 [based on the mapping results of NGS reads]) (38). The functional importance of the spike glycoprotein likely resulted in a conservative gene sequence. However, the abundant advantage of the master mutants may be larger than the true situation due to the potential amplification bias (39). Conversion events between the master and minor mutants can happen, such as the master mutant with F^1040^ and the public dominant strain with G^614^ (25, 26) may both exist as minor mutants in our early-infected samples (Fig. 3; see also Fig. S8). The conversion event may happen between the minor and master mutants when a minor mutant adapts to a specific high selective pressure (40). Variants with G^614^ were 2.6 to 9.3 times stronger than the early ancestor (41). Although mutants with the F^1040^ amino acid may obtain a fitness advantage (see Fig. S4 and 5), it had not spread as widely as the variants with G^614^, and it was only be found in strains from North America after ∼10 months (see Fig. S3 and Table S4 in the supplemental material). A timely clinical testing and isolation treatment may play a crucial role in blocking the spread of F^1040^ variants.

Except for F^1040^ and G^614^, more than 85% amino acid changes of the globally prevalent variants can be found in the quasispecies of early-infected patients (see Table S9), including the current predominant lineages such as B.1.1.7 (42) and B.1.351 (43) (Fig. 4). Quasispecies mutant spectra can be treated as the advantage mutation pool of the potential epidemic variants (future or current). Importantly, the mutation pool may contain potential fitness substitutions related to drugs or inhibitor resistance and antibody escape. The host cell-derived serine protease TMPRSS2 plays a key role in spike protein and ACE2 interaction (44); it is therefore taken as an important target for the known inhibitors, such as camostat mesilate (45). The predominant mutation D614G showed potential resistance to the camostat mesilate through enhancing affinity between the S1-S2 hinge region and TMPRSS2 protease (46). The spike receptor binding domain (RBD) is essential for viral entry by interacting with the ACE2 receptor on host cells and is the key target for neutralizing antibodies (47). Most of the candidate substitutions relating to antibody escape on the RBD of circulating SARS-CoV-2 in GISAID (48) could be found in the early-infected patients (see Table S8). The preexistence of potential fitness mutants suggests the importance of quasispecies on the development or selection of antiviral drugs.

The range of fitness mutations may be limited because most of mutations (∼65%) could be recurrent between hosts with different lineages and spread clusters (see Table S3). In this situation, the same advantage variation could exist in several unrelated hosts at the same time, which made it complicated to trace the virus origins. The predominant G^614^ genotype simultaneously existed in minor mutants of patients from two unrelated spread clusters (see Fig. S8). Under the possible conversion event, the minor mutants with G^614^ had the ability to become the master and further break through the transmission bottleneck. It was reasonable to assume that the epidemic variants with G^614^ were more likely multi-originated from different hosts. Results from the variation tracking had also shown a recurrent pattern of G^614^ increased at multiple geographic levels (26). The consensus genome sequences only reflected the master mutants and contained limited mutations (17–20), investigating the origins of a variant should consider the hidden dusty minor mutants.

Different sampling sites (tissues/organs) exhibited different pressures on virus survival and further shaped the fitness landscape of the quasispecies. Sputum samples showed a significantly lower complexity than did nasopharyngeal swabs and stools (Fig. 5a), where the fitness advantage of the master mutant was further amplified. On the contrary, stool samples had the largest quasispecies diversity (complex and genetic distance) than other two types (Fig. 5), where the fitness advantage of the master mutant was decreased slightly and the classes of mutants tended to be diversified. The diversifying of the quasispecies mutants indicated potential independent virus replications in different tissues or organs. Nasopharyngeal swab samples are from the upper respiratory tract, and sputum samples are from the lower respiratory tract. Different tissue types and spatial positions lead to differences in immune restriction, virus dynamics, and virus replication and then lead to diversification of quasispecies. Similar replication patterns had also been observed between throat and lung (49) or between the lower (induced sputum) and upper (nasopharyngeal swab) respiratory tracts (31, 34). No reports have demonstrated that viable SARS-CoV-2 virus can be directly isolated from stools. The stool SARS-CoV-2 viral RNA may be from swallowed discharges of the upper and lower respiratory tract (mixture), not from infected intestinal mucosa or bile ducts, which confers the larger diversity in stool samples than sputum or nasopharyngeal swab samples. The mixture of the virus may introduce more variations and further increase the diversity and genetic distance of the quasispecies.

Evolutionary analysis has shown that a continuous S2 region, including HR1, CH, and CD subdomains, was likely undergone a positive selection pressure (*dN/dS* > 1) (Fig. 6). It has been reported that the HR (membrane fusion related) combined with the RBDs played a crucial role in entering into the host cell and adapting to the immune system (50). At the consensus genome level, a potential positive selection site, such as G999C, had also been identified in the CH domain (51). Moreover, our results also suggested that the fusion peptide (FP) in the S2 region tended to remain fixed (*dN/dS* < 1 [purifying selection]). However, no selective signals were detected in the RBD. One possible reason is that the evolution period of quasispecies was short in the hosts. Nevertheless, highly selective pressures were acting on the S2 region, indicating the cell membrane fusion-related domains were likely crucial for the intrahost survival of SARS-CoV-2.

There are limitations in this study. First, due to technical limitations, it is currently difficult to obtain a haplotype with a full-length genome (∼29 kb), and the spike gene (∼4 kb) may partially reflect the genome-wide quasispecies information. Second, an insufficient number of samples and a short period of follow-up made it difficult for us to observe the dynamics on a relatively large scale. Third, due to the sequencing errors of the SMRT platform, the present study only focused on SNVs. Other mutation types, such as insertions, deletions, and structural variations, should also be explored in future studies.

Ultradeep sequencing of the spike gene facilitated our study of quasispecies with a relatively high resolution. We not only observed a stable quasispecies structure but also found a potential conversion event between the master and minor mutants. Mutation spectra of a host (especially the minor mutants) likely contained the dominant variations of the current or future epidemic strains. The prevalent variants might originate from multiple hosts. Cell membrane fusion-related domains of the spike glycoprotein revealed a high selective pressure and functional importance during intrahost evolution. Future molecular epidemiologic investigations, clinical interventions, and vaccine designs need to consider the SARS-CoV-2 quasispecies that are missed by routine single consensus genome.

## MATERIALS AND METHODS

### Participants in the study.

Nine patients with laboratory-confirmed COVID-19 were included in this study. All of them were admitted to Chongqing Public Health Medical Center (CPHMC), the designated hospital for COVID-19 treatment in the Chongqing center area. This study was approved by the CPHMC ethics committee (2020-002-01-KY) and conducted in accordance with Declaration of Helsinki principles. Written informed consent was obtained from each subject.

According to the epidemic history, all patients were divided into two single-source infected clusters or groups: the first group (FG) and the second group (SG) (Fig. 1a). The FG contained a familial cluster containing five SARS-CoV-2-infected patients, including one first-generation case infected in Wuhan (patient FG1), and four second-generation cases (FG2, FG3, FG4, and FG5) infected by FG1 in Chongqing, China (Fig. 1b). Patients directly infected by the first-generation patient were defined as second-generation patients. Similarly, third-generation patients were infected by second-generation patients. The first case FG1 (a 70-year-old male) was a local resident in Wuhan who came to Chongqing to visit his younger sister’s (FG2) family (FG2’s husband was FG3, FG2’s son was FG4, and FG2’s grandson was FG5) in 15 January 2020 by taking a train, and then they lived together from that day onward. On that day, FG1 presented a symptom of fever (highest temperature, 37.7°C) and coughing and took an antipyretic. FG1 went to a clinic on 21 January due to no relief of symptoms and tested positive for SARS-CoV-2 on January 24. FG1 was then transferred to the designated hospital (CPHMC). FG2 (60 years old), FG3 (62 years old), FG4 (36 years old), and FG5 (10 years old) began to experience present fever and/or cough symptoms, with highest temperatures of 39.3, 38, 37.5, and 38°C from 24, 19, 24, and 25 January, respectively. All second-generation patients were tested on 25 and 26 January; the results were positive, and they were transferred to CPHMC also on 27 and 31 January. CT scans showed classical diffuse ground-glass opacity in both sides of the lungs for all five patients, and they received lopinavir/ritonavir plus interferon-α antiviral treatment. In addition, FG1, FG2, and FG3 were given oxygen support though nasal cannula. FG1 developed severe illness with the underlying condition of 15-year hypertension. All of these patients recovered and were discharged from the hospital after treatment.

The SG contains four second- to third-generation patients infected by the same first-generation case, including one patient (SG1) from an unknown family and the remaining three patients from the same family (SG2, SG3, and SG4) (Fig. 1a). The first-generation cases returned to Chongqing from Wuhan on 20 January and then had dinner with two friends (SG1 and SG2) on 21 January. SG1 and SG2 presented with symptoms of fever and cough, took self-administered antipyretic, and received positive test results on 23, 24, and 26 January. Through direct contact, two family members of SG2 were also infected and revealed positive viral RNA results on 19 January, including SG2’s wife SG4 and SG4’s father SG3. According to the order of contact, SG1 and SG2 were considered as second-generation cases, while SG3 and SG4 were third-generation cases. For the patients from FG and SG, a total 14 samples were extracted from nasopharyngeal swab (NS), sputum (SP), or stool (ST). The sample time points are presented in Fig. 1b. All patients recovered and were discharged from the hospital after treatment.

### Extraction and amplification of the spike gene.

Viral RNA was extracted by using a EZ1 virus minikit (Qiagen, Germany), and then 8 μl of each was reverse transcribed into cDNA by using a commercial company’s reverse transcription kit (TaKaRa, 6110A). The full-length spike gene (3,882 nt) was further amplified by using a pair of RT-PCR primers designed by online methods from the National Center for Biotechnology Information. The forward primer was 5′-barcod-AGGGGTACTGCTGTTATGTCT-3′, and the reverse primer was 5′-barcod-GCGCGAACAAAATCTGAAGG-3′. We used a 50-μl PCR system to conduct the PCR, and the system included 5 μl of SARS-CoV-2 cDNA, 2.5 μl of forward and reverse primers (10 μmol/liter), 25 μl of Q5 High-Fidelity 2× Master Mix (NEB, M0492S), and complement volume with nuclease-free water (Thermo Fisher). The PCR conditions were as follows: denaturation, 98°C/30 s; circulation (35 times), 98°C/10 s, 54°C/30 s, and 72°C/3 min; and extension, 72°C/6 min. The PCR product was electrophoretic in 0.8% agarose gel to verify whether the product was successfully amplified. Next, we used AMPure PB beads (Pacific Biosciences, USA) for PCR product size selection. First, the AMPure PB beads were diluted to 35% using elution buffer; the diluted AMPure PB beads were then used for enrichment fragments larger than 3 kb (the target fragment was 3,882 nt), and the volume of the diluted AMPure PB beads was about three times the sample volume. After size selection, full-length DNA of the spike gene was quantified using a Qubit 2.0 fluorometer and a Qubit dsDNA HS assay kit (Thermo Fisher, Q32851). Due to sample quality or load of viral RNA, not all three sampling types successfully amplified the target segment. In addition, seven samples were obtained using next-generation sequencing reads from metagenomic sequencing, which was used to access the amplification bias by BWA software (52) (see Fig. S1). Finally, a total of 14 samples passed the quality control and were screened for further analysis (see Table S1). The sample names were generated using patient code numbers, the sampling date, and the sources of the nucleic acid. For example, “FG1-0126-NS” means viral RNA was extracted form nasopharyngeal swabs of FG1 patients on 26 January 2020.

### SMRT sequencing of the target segments.

About 100 ng of full-length DNA of spike gene was used for library construction. In short, the full-length cDNA was subjected to damage repair, end repair/A-tailing, and ligation of the SMRT adapter and unique label for each sample. The primers and DNA-binding polymerase were combined to generate a complete SMRT bell library. After qualitatively analyzing the library, a PacBio Sequel I platform was used for sequencing according to the effective concentration of the library and the data output requirements. We applied SMRT Link software package (https://www.pacb.com/support/software-downloads/ to obtain the CCSs), and only CCSs with more than five full passes were considered for the additional analysis.

### Data processing.

After performing the quality control and removing the low-quality subreads (minimum predicted accuracy of 90%), we obtained 26.97 Gb of subreads for all 14 samples (see Table S2). Our results showed that the gap number of CCSs decreased rapidly and tended to be a stable low level when the pass was equal to 5 (Fig. 2a). Further statistics showed that such reads account for ∼90% of total sequencing data (Fig. 2b; see also Table S2). The spike gene of strain Wuhan-Hu-1 (extracted from patients with early infection in Wuhan; GenBank accession number MN908947.3 [submitted on 5 January 2020]) (53) was used as reference to detect SNVs. We applied BLAST (54) software to perform an alignment between CCSs and the reference, and then we obtained all of the CCSs containing the full length of the spike gene (Table 1). Meanwhile, SNV information was extracted using PERL scripts based on the BLAST results. CCSs with the same haplotype were further clustered into the one mutant (haplotype), and its abundance was further calculated using PERL scripts (see Fig. S2). The software PopART (v4.8.4) was used to perform haplotype clustering (55). The diversity of quasispecies was detected by calculating the standardized Shannon entropy (Sn) and the mean genetic distance (Fig. 5). The former formula is Sn = –Σi (pi ⋅ lnpi)/ln*N*, where pi is the frequency of the top abundance quasispecies, and *N* is the total number of quasispecies haplotypes. The R program was applied to perform the principal component analysis (see Fig. S6). The three-dimensional ribbon models for the spike protein were prepared by UCSF Chimera (56) based on the entry 6VSB in Protein Data Bank (57) (see Fig. S4 and S5). MEGAX (58) was used to calculate the mean genetic distance and to carry out the phylogenetic analysis. For haplotype clustering, more than 68,000 public consensus genomes of SARS-CoV-2 were collected from the GenBank database by 1 April 2020. To query SNV 3118G→T, an online BLAST tool was used to align the master mutant to the consensus genomes from the GenBank database. The genomes of the epidemic lineages of B.1.1.7, B.1.248, B.1.351, B.1.526, B.1.525, and B.1.429+427 were collected from GISAID database (as of 2 April 2020) (see Table S7 in the supplemental material). Meanwhile, all accumulated amino acid variations of the spike gene were also downloaded from GISAID database (as of 2 April 2020), and all 35 potential antibody escape mutations were collected from the literatures (see Tables S8 and S9). The mutations were annotated and extracted by using in-house PERL scripts.

### Data availability.

All the quasispecies amino acid variations and other data are included in the supplemental material online or available from the authors upon reasonable requests. Public SARS-CoV-2 consensus genomes are available in GenBank (https://www.ncbi.nlm.nih.gov/datasets/coronavirus/genomes/). All potential dominant amino acid variations (occurrence ≥ 10) can be found in the GISAID database. Please visit and log in to the official website https://www.gisaid.org/. The amino acid substitutions of the Spike gene can be accessed by clicking the “spike glycoprotein mutation surveillance” entry under the EpiCov item. Six epidemic variants are available by querying the lineage keywords of B.1.1.7, B.1.248, B.1.351, B.1.526, B.1.525, and B.1.429+427 at the “Search” entry under the EpiCov item.

All raw mutant sequences of 14 samples have been deposited in the National Genomics Data Center, Beijing Institute of Genomics, Chinese Academy of Sciences/China National Center for Bioinformation, under the accession numbers GWHBDHM00000000, GWHBDHN00000000, GWHBDHO00000000, GWHBDHP00000000, GWHBDHC00000000, GWHBDHD00000000, GWHBDHE00000000, GWHBDHF00000000, GWHBDHG00000000, GWHBDHH00000000, GWHBDHI00000000, GWHBDHJ00000000, GWHBDHK00000000, and GWHBDHL00000000, which are publicly accessible at https://ngdc.cncb.ac.cn/gwh/. The data can also be accessed under the accession number CRA004527 that is publicly accessible at https://ngdc.cncb.ac.cn/gsa/browse/CRA004527.
