# Supplementary material for: SARS-CoV-2 Quasispecies Provides an Advantage Mutation Pool for the Epidemic Variants
Source: Microbiol Spectr. 2021 Aug 4;9(1):10.1128/spectrum.00261-21. doi: 10.1128/spectrum.00261-21 (PMC8552775; doi:10.1128/spectrum.00261-21)
Supplement: SUPPLEMENTAL FILE 2 — Supplemental material. Download SPECTRUM00261-21_Supp_2_seq11.pdf, PDF file, 3.1 MB [file spectrum00261-21_supp_2_seq11.pdf]

Supplementary figures

Contents

Supplementary figure legends 2

Figure S1 Evaluating the mutation rate of the primers.. 6

Figure S2 Details of the top 30 abundant mutants of each sample... 21

Figure S3 The occurrence statistics of the consensus genomes with 3118G>T in Genbank database (by March 2021). 22

Figure S4 The position of 3118G>T on the spike protein (3D ribbon structure). 22

Figure S5 Simulation for the amino acid changes of the 3118 G>T. 23

Figure S6 Principal component analysis for all samples. 23

Figure S7 Occurrence of three hub spike haplotypes of Genbank in the quasispecies mutants. 24

Figure S8 Detecting a currently predominant SNV 1841A>G (D614G) in the minor mutants of the quasispecies. 24

Figure S9 Box plots of the nonsense and missense mutation rate for three sampling sites..... 25

Figure S10 Box plots of the dN and dS value. 25

Supplementary figure legends

Figure S1 Evaluating the mutation rate of the primers. The upper part shows the frequency distribution of the four bases. The middle represents the major allele. The lower part shows the depth of random NGS sequencing. More than 98% virus RNA contained the identical sequences with the amplification primers of AGGGGTACTGCTGTTATGTCT and CCTTCAGATTTGTTCGCGC.

Figure S2 Details of the top 30 abundant mutants of each sample. Each line represented a kind of mutant, green lines were the missense mutations and the red colors were synonymous mutations. The top rectangle represents the cumulative variations from quasispecies mutants. Functional domain of the spike gene were marked with different colors: NTD, N-terminal domain; RBD, receptor-binding domain; S1/S2, protease cleavage site; FP, fusion peptide; HR1, heptad repeat 1; CH, central helix; CD, connector domain; HR2, heptad repeat 2; TM, transmembrane domain; CT, cytoplasmic tail. The rightmost bar plots showed the proportion of mutants (abundance). Results of haplotype clustering were listed at the right bottom, where mutation spectrums centered by the master one were identified.

Figure S3 The occurrence statistics of the consensus genomes with 3118G>T in Genbank database (by March 2021). The results showed that 3118G>T was never emerged before October 2020 that was 10 month after the sampling date of the current study.

Figure S4 The position of 3118G>T on the spike protein (3D ribbon structure). The corresponding amino acid is highlighted by a red line and some potential functional domains are marked with arrow. The nucleotide of 3118T was located in the turn site between CH and CD sub-domains.

Figure S5 Simulation for the amino acid changes of the 3118 G>T. Part **a** showed the nucleotide of SG master mutant G (same with the early ancestor strain), and part **b** showed the nucleotide of FG master mutant T. The location of variation site was highlighted by yellow color, the changed angle of R-group was simulated by Chimera with the Posterior probability=0.30.

Figure S6 Principal component analysis for all samples. (**a**) Two clusters were identified based on all SNVs of the top 30 abundant mutants. (**b**) The SNV 3118T>G as the first dimension could fully distinguish the two single-source infection groups and made more than 98% contributions to the grouping.

Figure S7 Occurrence of three hub spike haplotypes of Genbank in the quasispecies mutants. Three haplotypes reflected for three master mutants within patients, and all these master mutants had already existed in the minor mutants of early infected patients.

Figure S8 Detecting a currently predominant SNV 1841A>G (D614G) in the minor mutants of the quasispecies. The nucleotide 1841G (amino acid G614) had already existed in 19 minor mutants from 4 early infected patients.

Figure S9 Box plots of the nonsense and missense mutation rate for three sampling sites. Both percentage of nonsense and missense mutation showed obviously higher in samples of stools. The mean values were marked on the figure.

Figure S10 Box plots of the dN and dS value. The mean dS values of each domain are almost the same (**a**), while the mean dN values of three domains of FP, HR1, and CD were significantly larger than the whole gene level (t-test) (**b**).

FG1-0126-SP: Average Major Allele Frequency 0.9454±0.0382

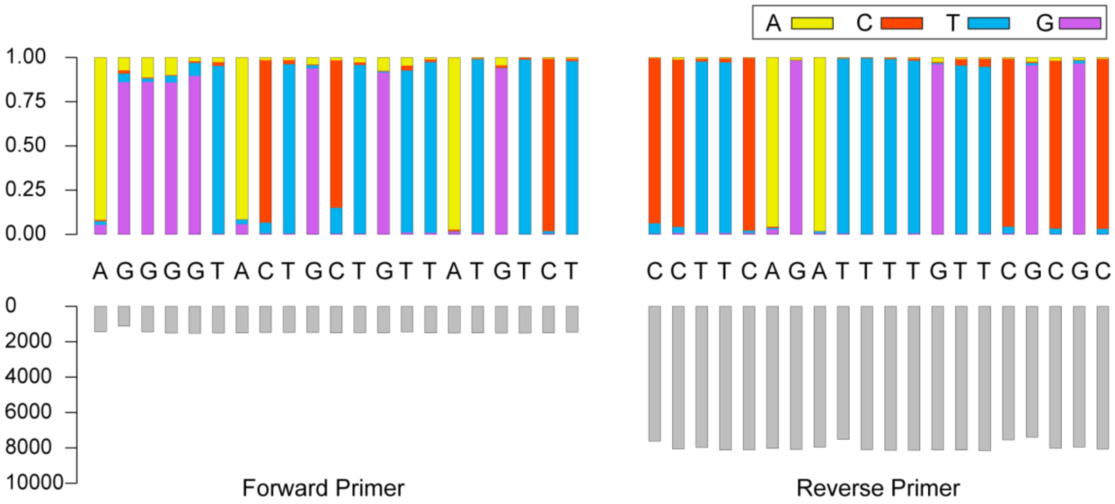

FG1-0129-NS: Average Major Allele Frequency 1.00

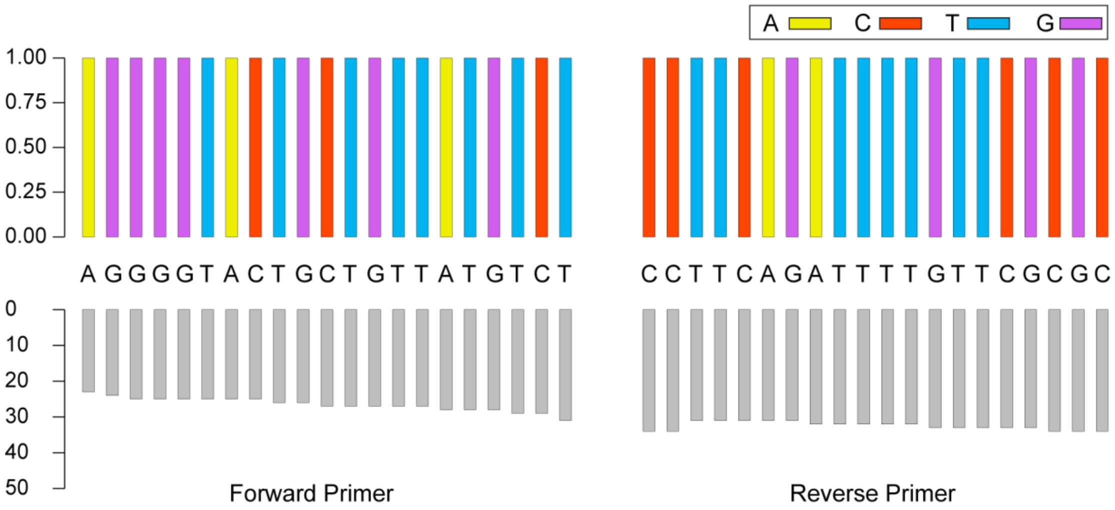

FG2-0127-NS: Average Major Allele Frequency 0.99±0.01

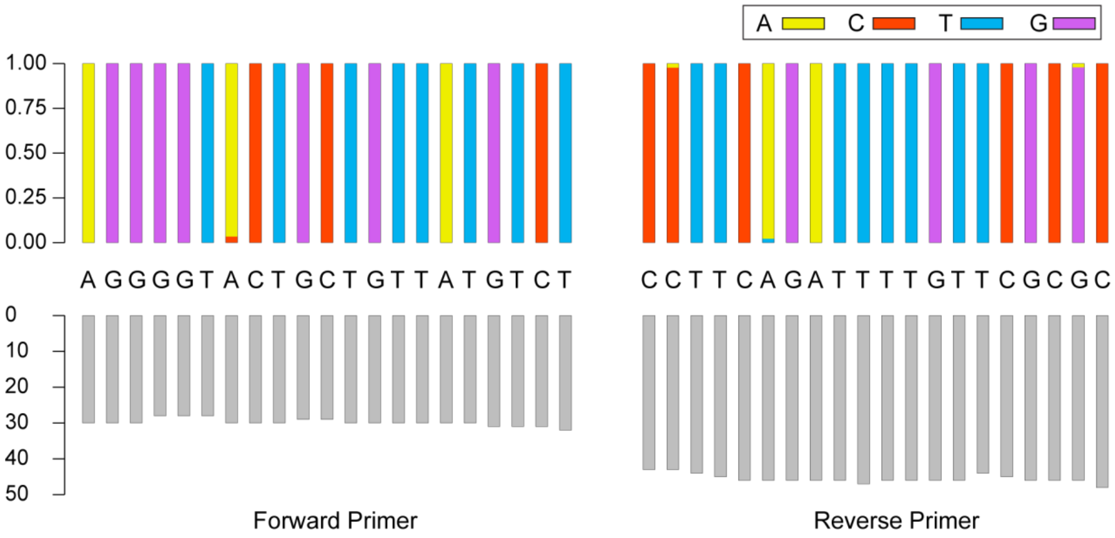

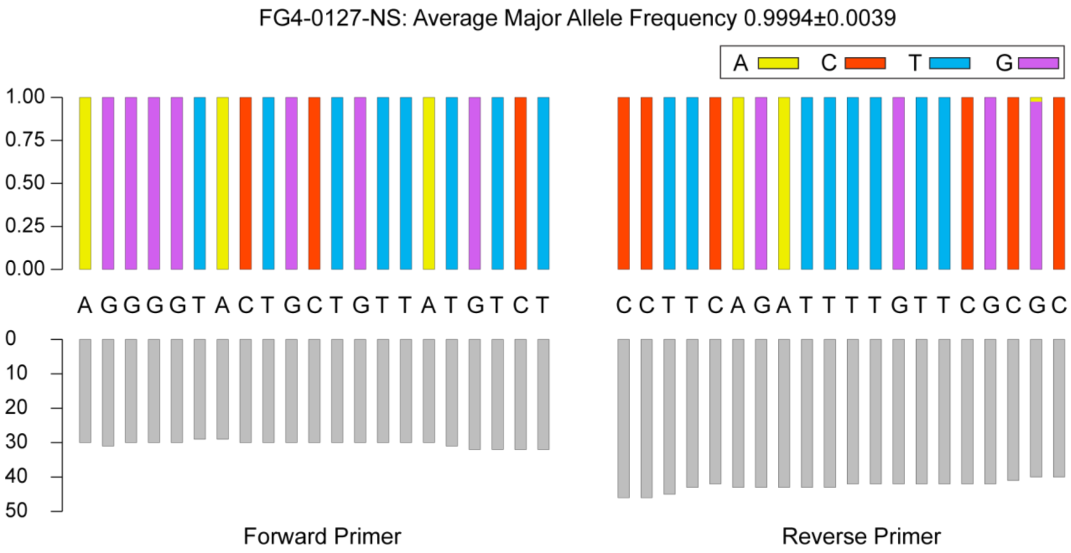
FG4-0127-NS: Average Major Allele Frequency 0.9994±0.0039
A C T G
1.00
0.75
0.50
0.25
0.00
A G G G G T A C T G C T G T T A T G T C T
C C T T C A G A T T T T G T T C G C G C
0
10
20
30
40
50
Forward Primer
Reverse Primer

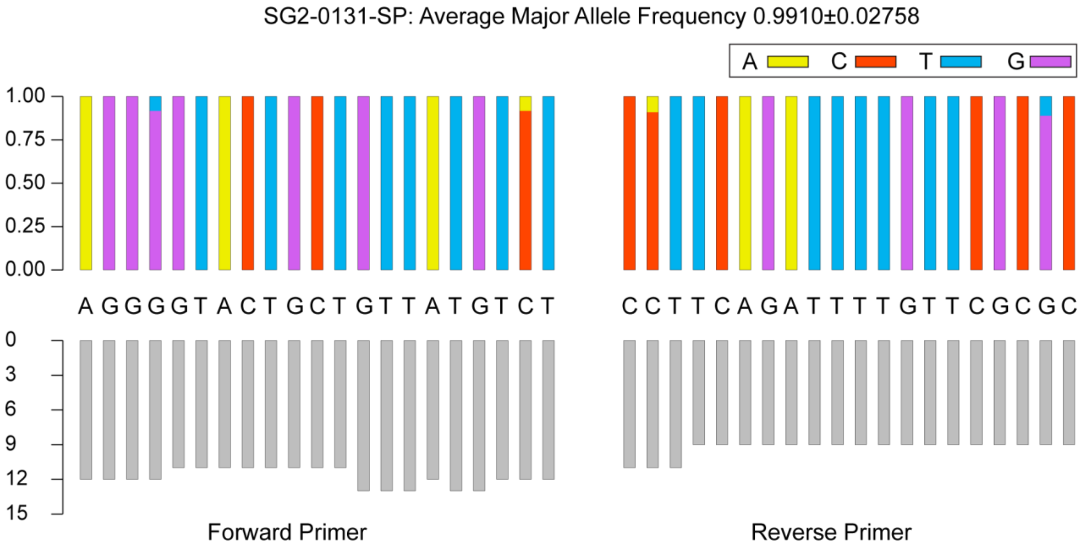
SG2-0131-SP: Average Major Allele Frequency 0.9910±0.02758
A C T G
1.00
0.75
0.50
0.25
0.00
A G G G G T A C T G C T G T T A T G T C T
C C T T C A G A T T T T G T T C G C G C
0
3
6
9
12
15
Forward Primer
Reverse Primer

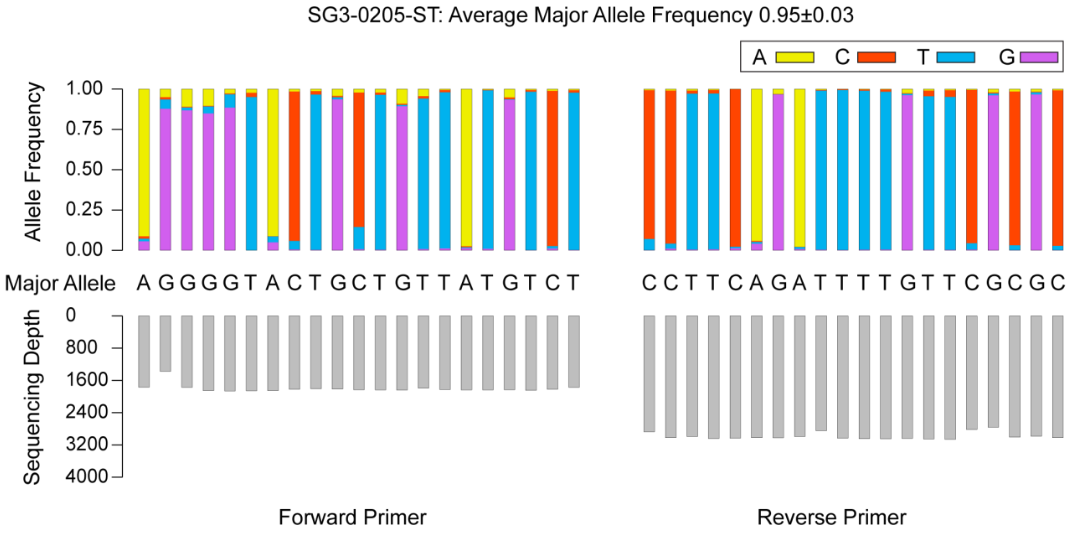
SG3-0205-ST: Average Major Allele Frequency 0.95±0.03
A C T G
Allele Frequency
1.00
0.75
0.50
0.25
0.00
Major Allele
A G G G G T A C T G C T G T T A T G T C T
C C T T C A G A T T T T G T T C G C G C
Sequencing Depth
0
800
1600
2400
3200
4000
Forward Primer
Reverse Primer

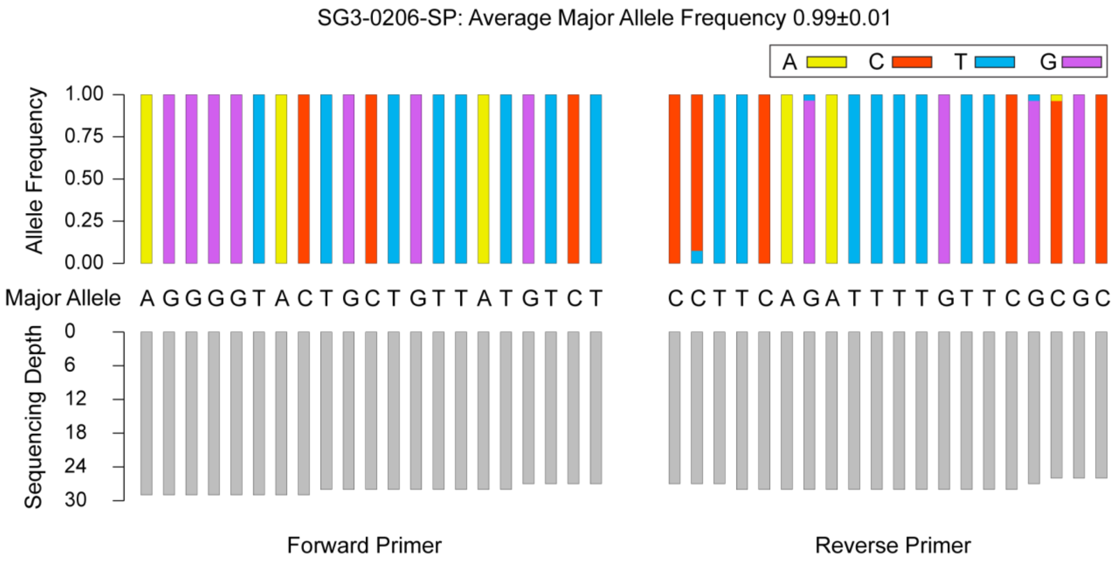

Figure S1 Evaluating the mutation rate of the primers. The upper part shows the frequency distribution of the four bases. The middle represents the major allele. The lower part shows the depth of random NGS sequencing. More than 98% virus RNA contained the identical sequences with the amplification primers of AGGGGTACTGCTGTTATGTCT and CCTTCAGATTTGTTCGCGC.

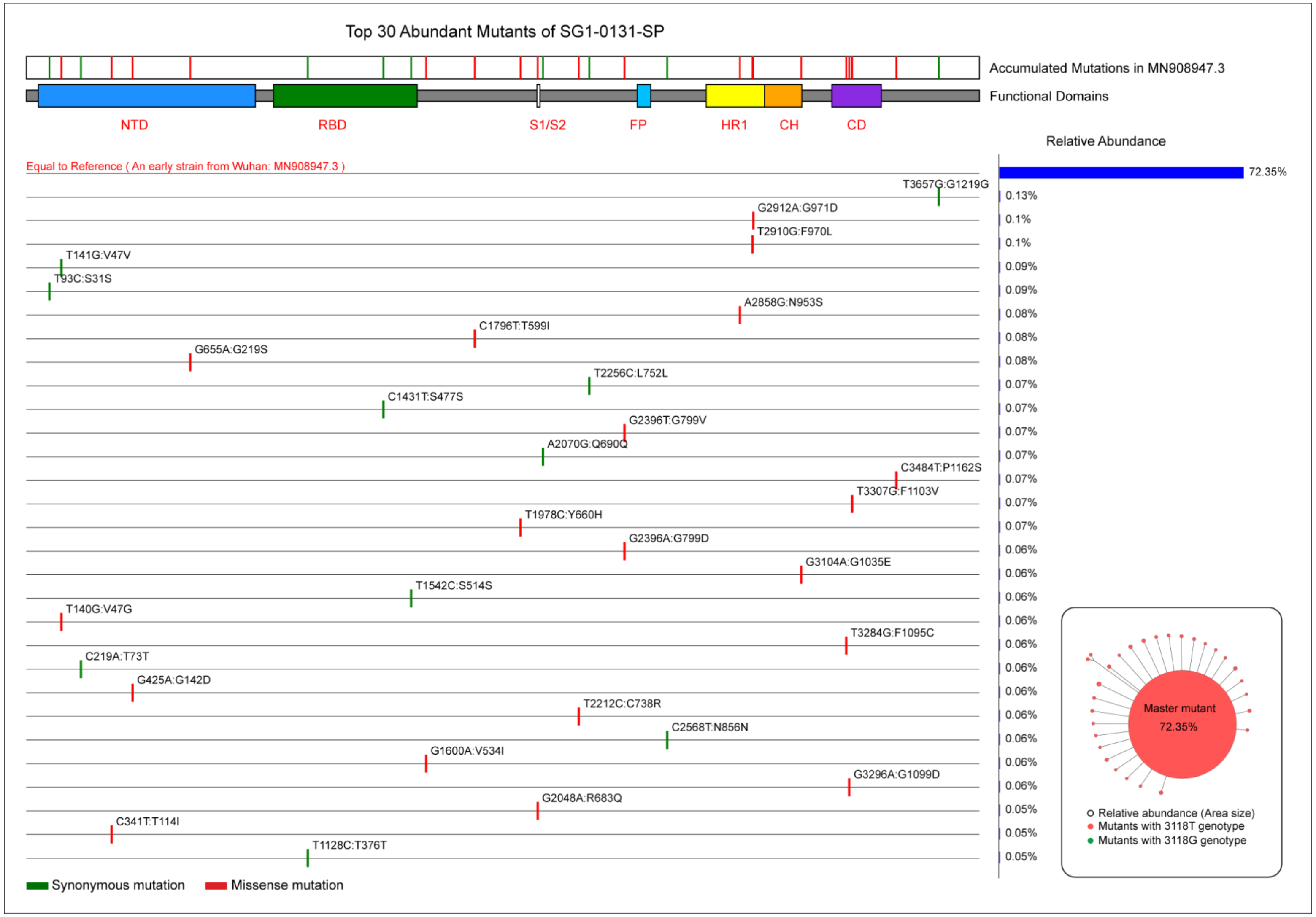

Top 30 Abundant Mutants of SG1-0131-SP
Accumulated Mutations in MN908947.3
Functional Domains
NTD
RBD
S1/S2
FP
HR1
CH
CD
Relative Abundance
Equal to Reference (An early strain from Wuhan: MN908947.3)
72.35%
T3657G:G1219G
0.13%
G2912A:G971D
0.1%
T2910G:F970L
0.1%
T141G:V47V
0.09%
T93C:S31S
0.09%
A2858G:N953S
0.08%
C1796T:T599I
0.08%
G655A:G219S
0.08%
T2256C:L752L
0.07%
C1431T:S477S
0.07%
G2396T:G799V
0.07%
A2070G:Q690Q
0.07%
C3484T:P1162S
0.07%
T3307G:F1103V
0.07%
T1978C:Y660H
0.07%
G2396A:G799D
0.06%
G3104A:G1035E
0.06%
T1542C:S514S
0.06%
T140G:V47G
0.06%
T3284G:F1095C
0.06%
C219A:T73T
0.06%
G425A:G142D
0.06%
T2212C:C738R
0.06%
C2568T:N856N
0.06%
G1600A:V534I
0.06%
G3296A:G1099D
0.06%
G2048A:R683Q
0.05%
C341T:T114I
0.05%
T1128C:T376T
0.05%
Master mutant
72.35%
Relative abundance (Area size)
Mutants with 3118T genotype
Mutants with 3118G genotype
Synonymous mutation
Missense mutation

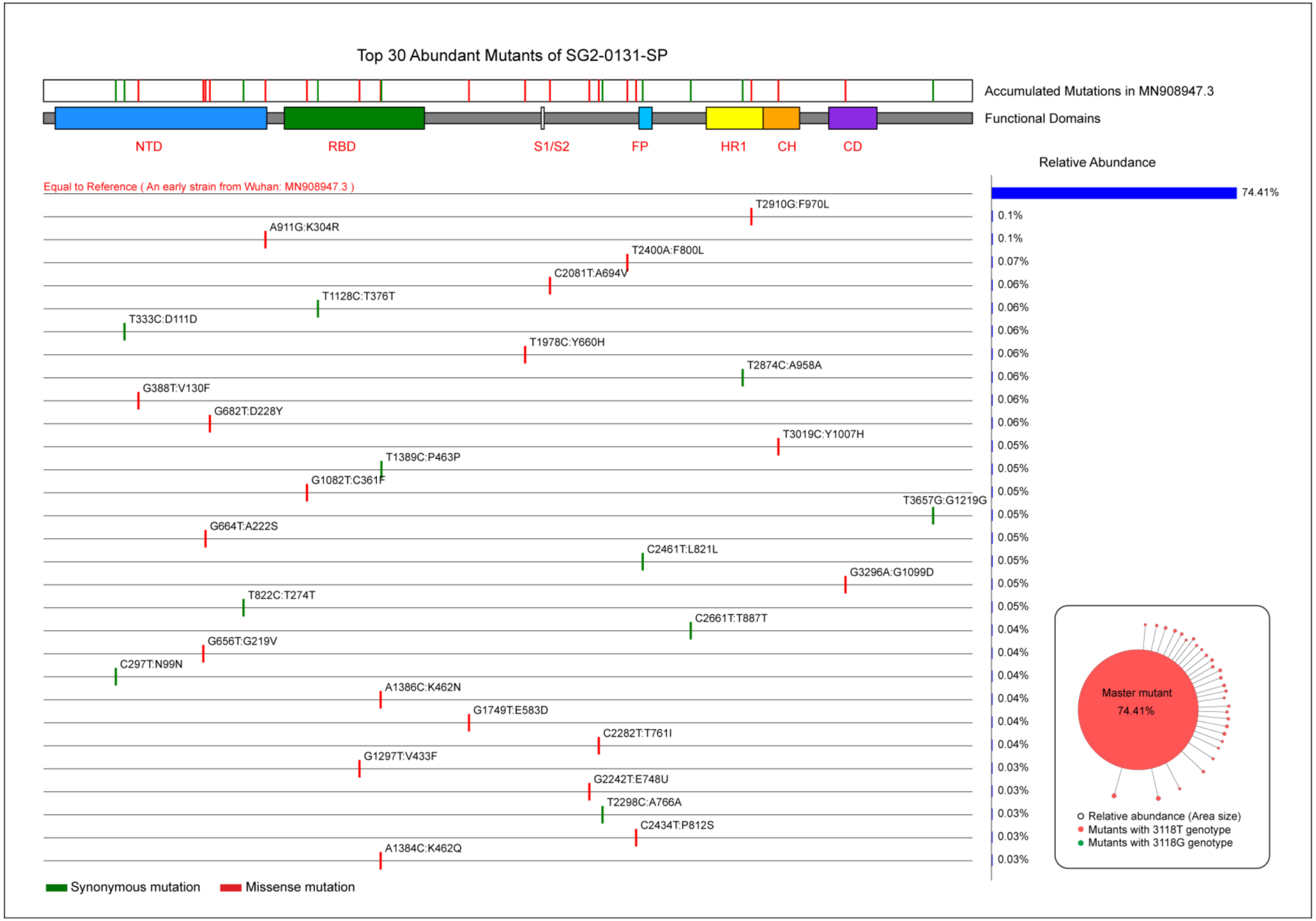
Top 30 Abundant Mutants of SG2-0131-SP
Accumulated Mutations in MN908947.3
Functional Domains
NTD
RBD
S1/S2
FP
HR1
CH
CD
Relative Abundance
Equal to Reference (An early strain from Wuhan: MN908947.3)
74.41%
T2910G:F970L
0.1%
A911G:K304R
0.1%
T2400A:F800L
0.07%
C2081T:A694V
0.06%
T1128C:T376T
0.06%
T333C:D111D
0.06%
T1978C:Y660H
0.06%
T2874C:A958A
0.06%
G388T:V130F
0.06%
G682T:D228Y
0.06%
T3019C:Y1007H
0.05%
T1389C:P463P
0.05%
G1082T:C361F
0.05%
T3657G:G1219G
0.05%
G664T:A222S
0.05%
C2461T:L821L
0.05%
G3296A:G1099D
0.05%
T822C:T274T
0.05%
C2661T:T887T
0.04%
G656T:G219V
0.04%
C297T:N99N
0.04%
A1386C:K462N
0.04%
G1749T:E583D
0.04%
C2282T:T761I
0.04%
G1297T:V433F
0.03%
G2242T:E748U
0.03%
T2298C:A766A
0.03%
C2434T:P812S
0.03%
A1384C:K462Q
0.03%
Master mutant
74.41%
Relative abundance (Area size)
Mutants with 3118T genotype
Mutants with 3118G genotype
Synonymous mutation
Missense mutation

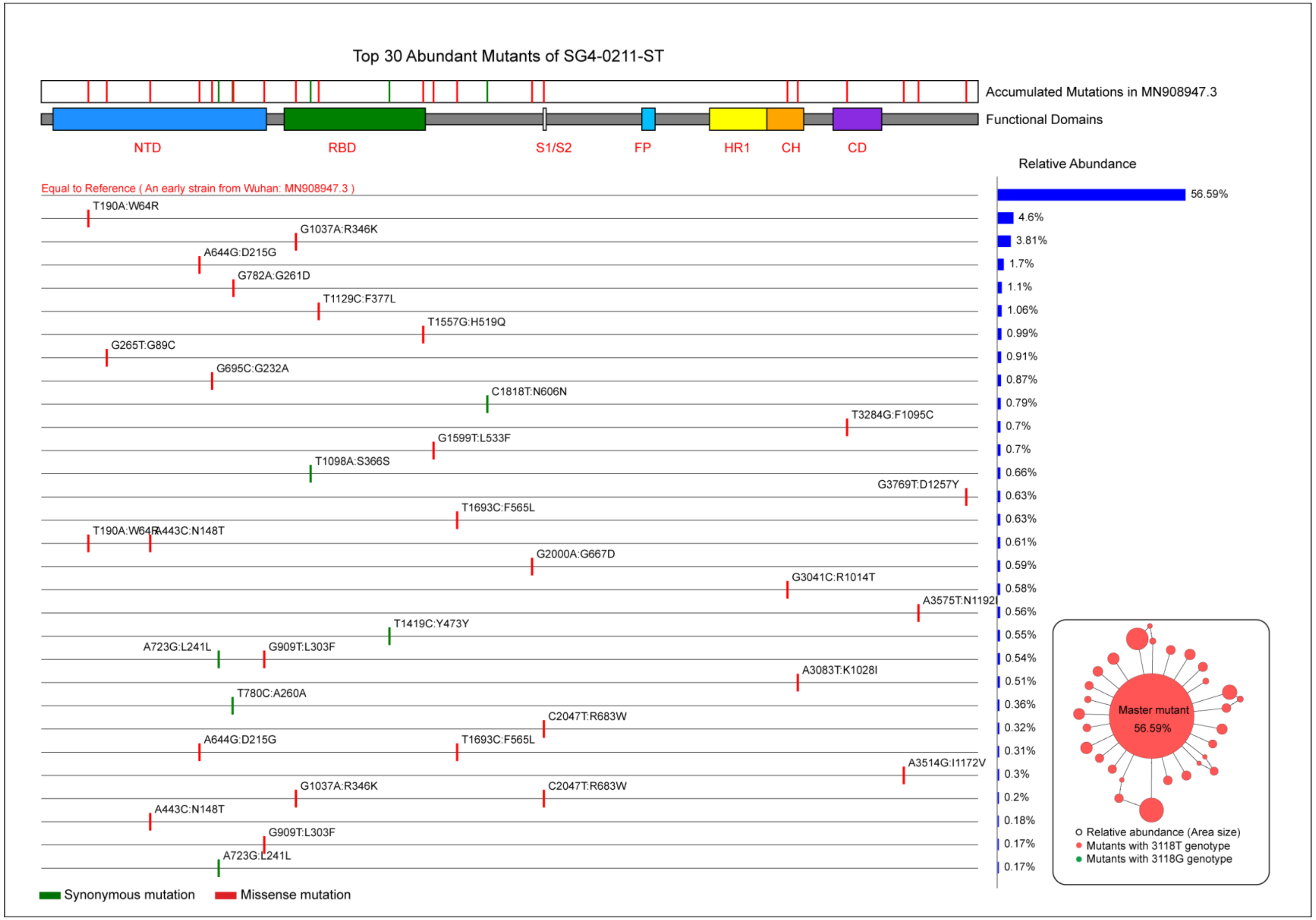

Top 30 Abundant Mutants of SG4-0211-ST
Accumulated Mutations in MN908947.3
Functional Domains
NTD
RBD
S1/S2
FP
HR1
CH
CD
Relative Abundance
Equal to Reference (An early strain from Wuhan: MN908947.3)
56.59%
T190A:W64R
4.6%
G1037A:R346K
3.81%
A644G:D215G
1.7%
G782A:G261D
1.1%
T1129C:F377L
1.06%
T1557G:H519Q
0.99%
G265T:G89C
0.91%
G695C:G232A
0.87%
C1818T:N606N
0.79%
T3284G:F1095C
0.7%
G1599T:L533F
0.7%
T1098A:S366S
0.66%
G3769T:D1257Y
0.63%
T1693C:F565L
0.63%
T190A:W64R
A443C:N148T
0.61%
G2000A:G667D
0.59%
G3041C:R1014T
0.58%
A3575T:N1192I
0.56%
T1419C:Y473Y
0.55%
A723G:L241L
G909T:L303F
0.54%
A3083T:K1028I
0.51%
T780C:A260A
0.36%
C2047T:R683W
0.32%
A644G:D215G
T1693C:F565L
0.31%
A3514G:I1172V
0.3%
G1037A:R346K
C2047T:R683W
0.2%
A443C:N148T
0.18%
G909T:L303F
0.17%
A723G:L241L
0.17%
Master mutant
56.59%
Relative abundance (Area size)
Mutants with 3118T genotype
Mutants with 3118G genotype
Synonymous mutation
Missense mutation

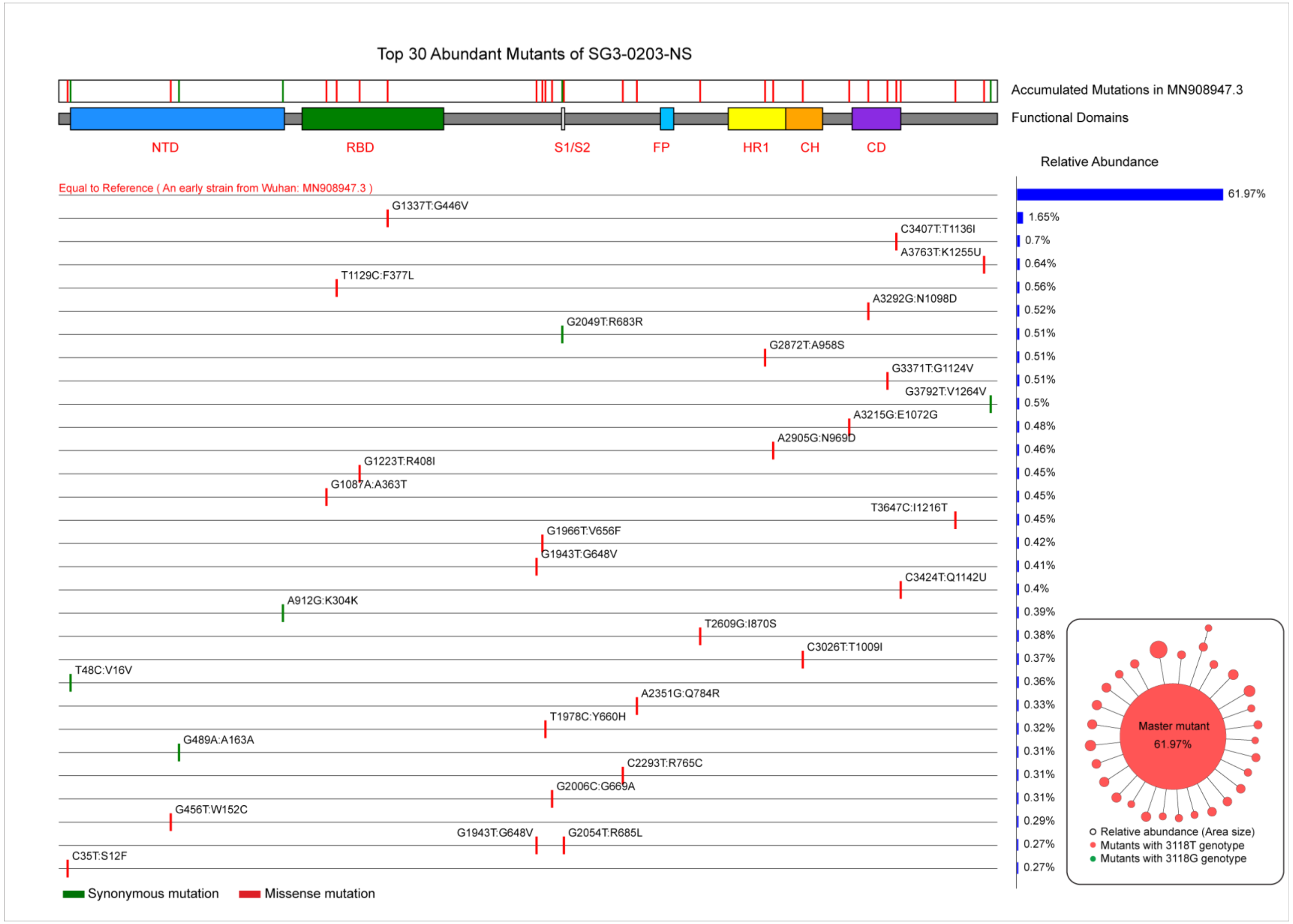

Top 30 Abundant Mutants of SG3-0203-NS
Accumulated Mutations in MN908947.3
Functional Domains
NTD
RBD
S1/S2
FP
HR1
CH
CD
Relative Abundance
Equal to Reference (An early strain from Wuhan: MN908947.3)
61.97%
G1337T:G446V
1.65%
C3407T:T1136I
0.7%
A3763T:K1255U
0.64%
T1129C:F377L
0.56%
A3292G:N1098D
0.52%
G2049T:R683R
0.51%
G2872T:A958S
0.51%
G3371T:G1124V
0.51%
G3792T:V1264V
0.5%
A3215G:E1072G
0.48%
A2905G:N969D
0.46%
G1223T:R408I
0.45%
G1087A:A363T
0.45%
T3647C:I1216T
0.45%
G1966T:V656F
0.42%
G1943T:G648V
0.41%
C3424T:Q1142U
0.4%
A912G:K304K
0.39%
T2609G:I870S
0.38%
C3026T:T1009I
0.37%
T48C:V16V
0.36%
A2351G:Q784R
0.33%
T1978C:Y660H
0.32%
G489A:A163A
0.31%
C2293T:R765C
0.31%
G2006C:G669A
0.31%
G456T:W152C
0.29%
G1943T:G648V
G2054T:R685L
0.27%
C35T:S12F
0.27%
Master mutant
61.97%
Relative abundance (Area size)
Mutants with 3118T genotype
Mutants with 3118G genotype
Synonymous mutation
Missense mutation

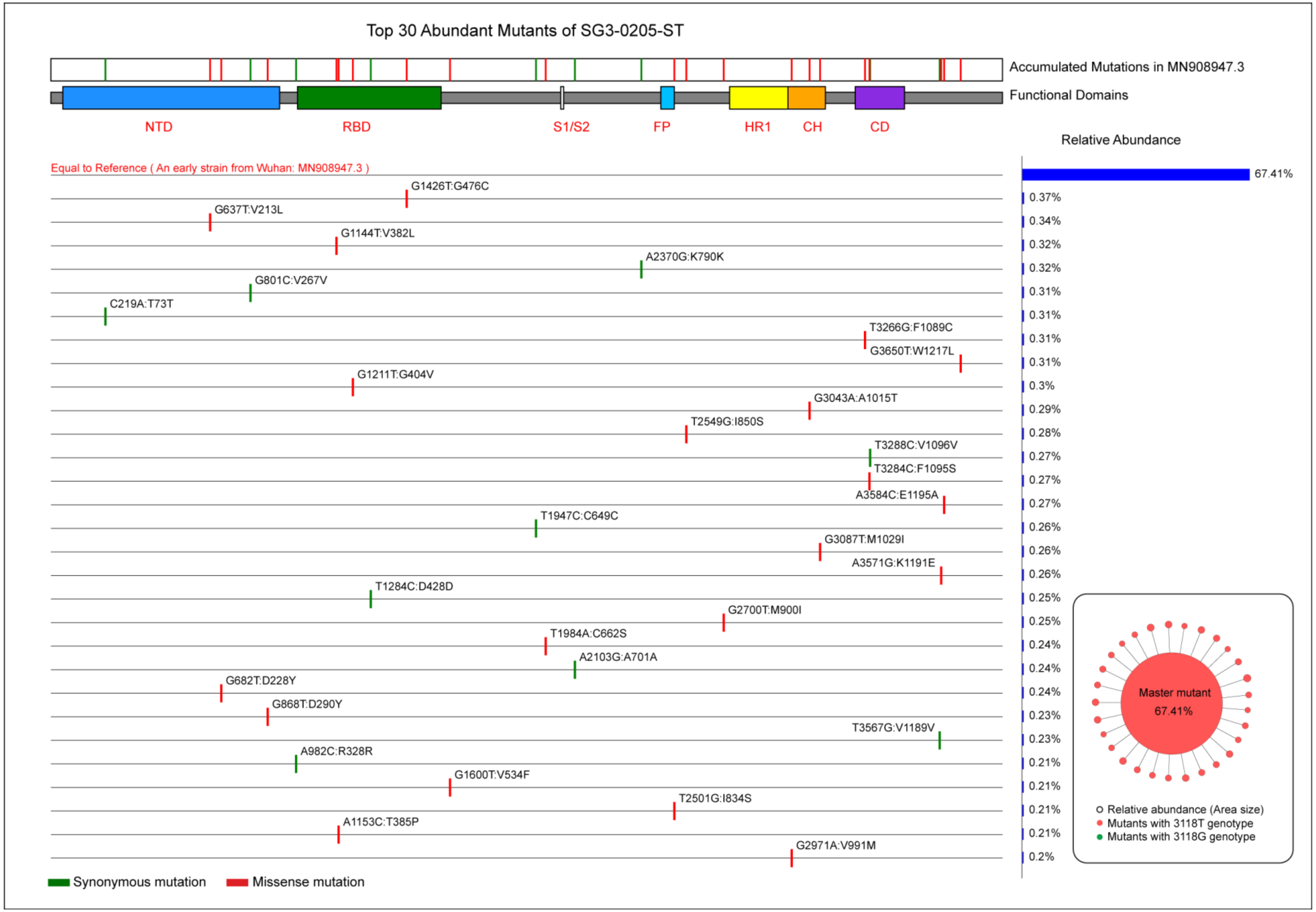

Top 30 Abundant Mutants of SG3-0205-ST
Accumulated Mutations in MN908947.3
Functional Domains
NTD
RBD
S1/S2
FP
HR1
CH
CD
Relative Abundance
Equal to Reference (An early strain from Wuhan: MN908947.3)
67.41%
G1426T:G476C
0.37%
G637T:V213L
0.34%
G1144T:V382L
0.32%
A2370G:K790K
0.32%
G801C:V267V
0.31%
C219A:T73T
0.31%
T3266G:F1089C
0.31%
G3650T:W1217L
0.31%
G1211T:G404V
0.3%
G3043A:A1015T
0.29%
T2549G:I850S
0.28%
T3288C:V1096V
0.27%
T3284C:F1095S
0.27%
A3584C:E1195A
0.27%
T1947C:C649C
0.26%
G3087T:M1029I
0.26%
A3571G:K1191E
0.26%
T1284C:D428D
0.25%
G2700T:M900I
0.25%
T1984A:C662S
0.24%
A2103G:A701A
0.24%
G682T:D228Y
0.24%
G868T:D290Y
0.23%
T3567G:V1189V
0.23%
A982C:R328R
0.21%
G1600T:V534F
0.21%
T2501G:I834S
0.21%
A1153C:T385P
0.21%
G2971A:V991M
0.2%
Master mutant
67.41%
Relative abundance (Area size)
Mutants with 3118T genotype
Mutants with 3118G genotype
Synonymous mutation
Missense mutation

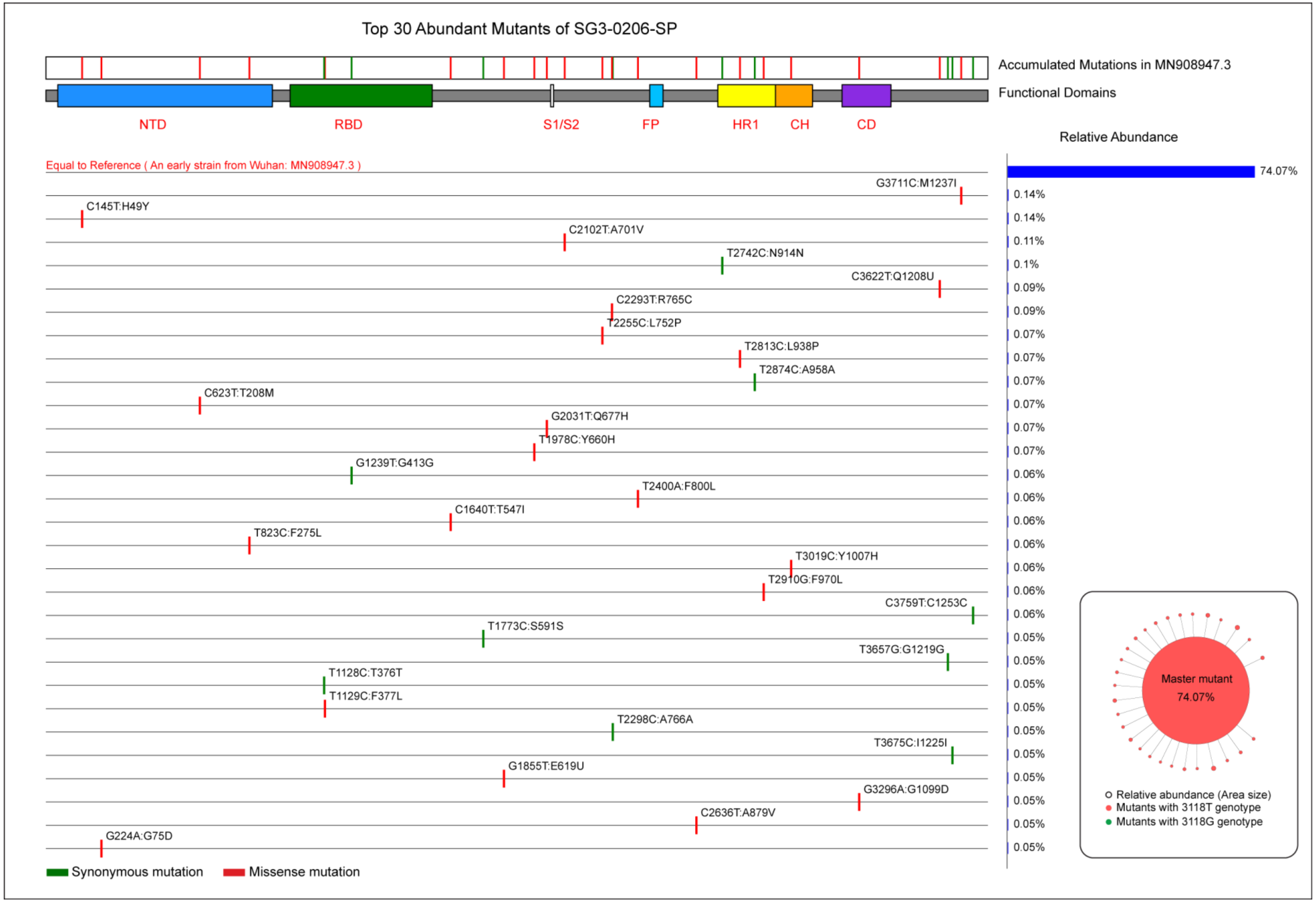
Top 30 Abundant Mutants of SG3-0206-SP
Accumulated Mutations in MN908947.3
Functional Domains
NTD
RBD
S1/S2
FP
HR1
CH
CD
Relative Abundance
Equal to Reference (An early strain from Wuhan: MN908947.3)
74.07%
G3711C:M1237I
0.14%
C145T:H49Y
0.14%
C2102T:A701V
0.11%
T2742C:N914N
0.1%
C3622T:Q1208U
0.09%
C2293T:R765C
0.09%
T2255C:L752P
0.07%
T2813C:L938P
0.07%
T2874C:A958A
0.07%
C623T:T208M
0.07%
G2031T:Q677H
0.07%
T1978C:Y660H
0.07%
G1239T:G413G
0.06%
T2400A:F800L
0.06%
C1640T:T547I
0.06%
T823C:F275L
0.06%
T3019C:Y1007H
0.06%
T2910G:F970L
0.06%
C3759T:C1253C
0.06%
T1773C:S591S
0.05%
T3657G:G1219G
0.05%
T1128C:T376T
0.05%
T1129C:F377L
0.05%
T2298C:A766A
0.05%
T3675C:I1225I
0.05%
G1855T:E619U
0.05%
G3296A:G1099D
0.05%
C2636T:A879V
0.05%
G224A:G75D
0.05%
Master mutant
74.07%
Relative abundance (Area size)
Mutants with 3118T genotype
Mutants with 3118G genotype
Synonymous mutation
Missense mutation

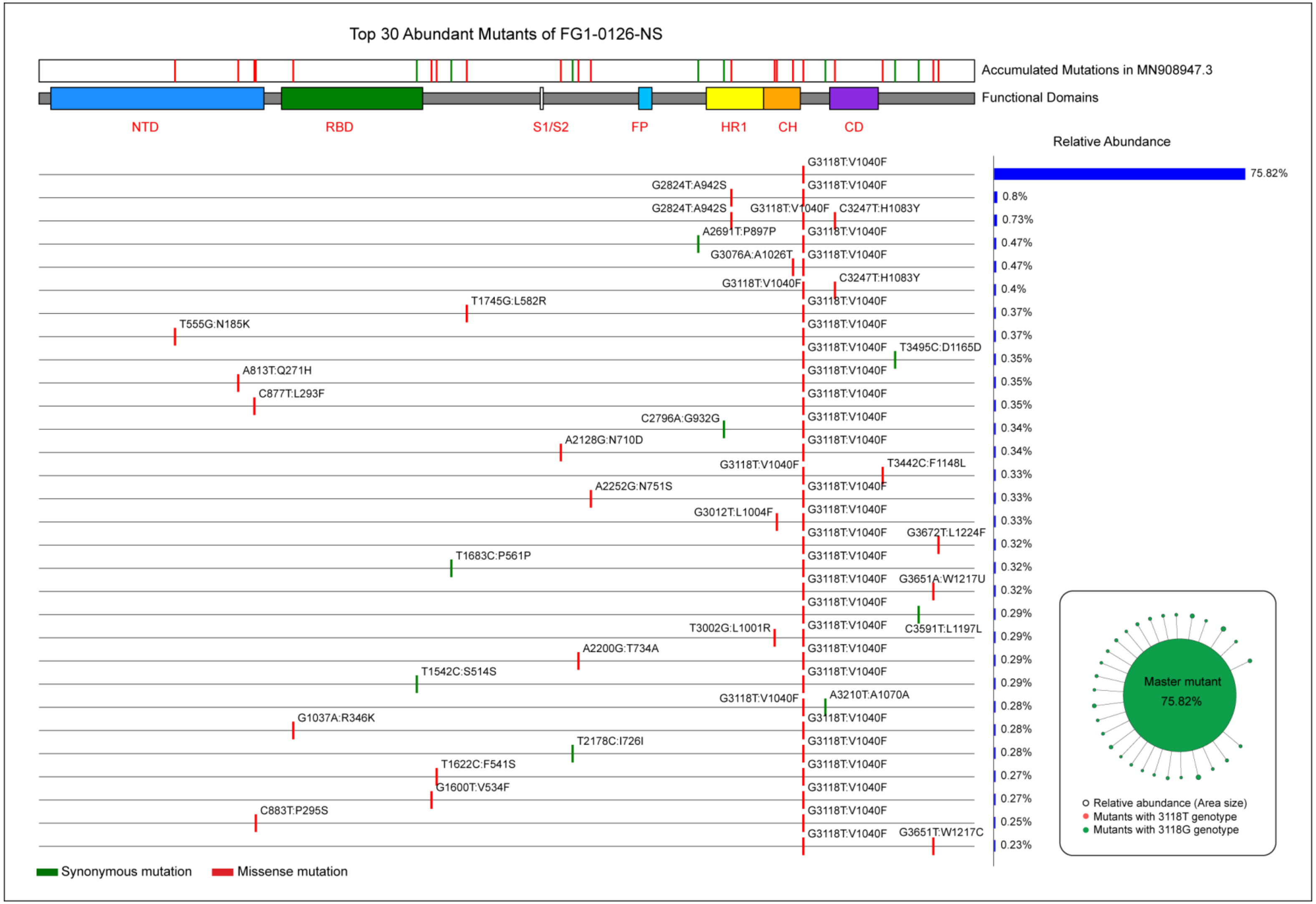
Top 30 Abundant Mutants of FG1-0126-NS
Accumulated Mutations in MN908947.3
Functional Domains
NTD
RBD
S1/S2
FP
HR1
CH
CD
Relative Abundance
G3118T:V1040F
75.82%
G2824T:A942S
G3118T:V1040F
0.8%
G2824T:A942S
G3118T:V1040F
C3247T:H1083Y
0.73%
A2691T:P897P
G3118T:V1040F
0.47%
G3076A:A1026T
G3118T:V1040F
0.47%
G3118T:V1040F
C3247T:H1083Y
0.4%
T1745G:L582R
G3118T:V1040F
0.37%
T555G:N185K
G3118T:V1040F
0.37%
G3118T:V1040F
T3495C:D1165D
0.35%
A813T:Q271H
G3118T:V1040F
0.35%
C877T:L293F
G3118T:V1040F
0.35%
C2796A:G932G
G3118T:V1040F
0.34%
A2128G:N710D
G3118T:V1040F
0.34%
G3118T:V1040F
T3442C:F1148L
0.33%
A2252G:N751S
G3118T:V1040F
0.33%
G3012T:L1004F
G3118T:V1040F
0.33%
G3118T:V1040F
G3672T:L1224F
0.32%
T1683C:P561P
G3118T:V1040F
0.32%
G3118T:V1040F
G3651A:W1217U
0.32%
G3118T:V1040F
C3591T:L1197L
0.29%
T3002G:L1001R
G3118T:V1040F
0.29%
A2200G:T734A
G3118T:V1040F
0.29%
T1542C:S514S
G3118T:V1040F
0.29%
G3118T:V1040F
A3210T:A1070A
0.28%
G1037A:R346K
G3118T:V1040F
0.28%
T2178C:I726I
G3118T:V1040F
0.28%
T1622C:F541S
G3118T:V1040F
0.27%
G1600T:V534F
G3118T:V1040F
0.27%
C883T:P295S
G3118T:V1040F
0.25%
G3118T:V1040F
G3651T:W1217C
0.23%
Master mutant
75.82%
Relative abundance (Area size)
Mutants with 3118T genotype
Mutants with 3118G genotype
Synonymous mutation
Missense mutation

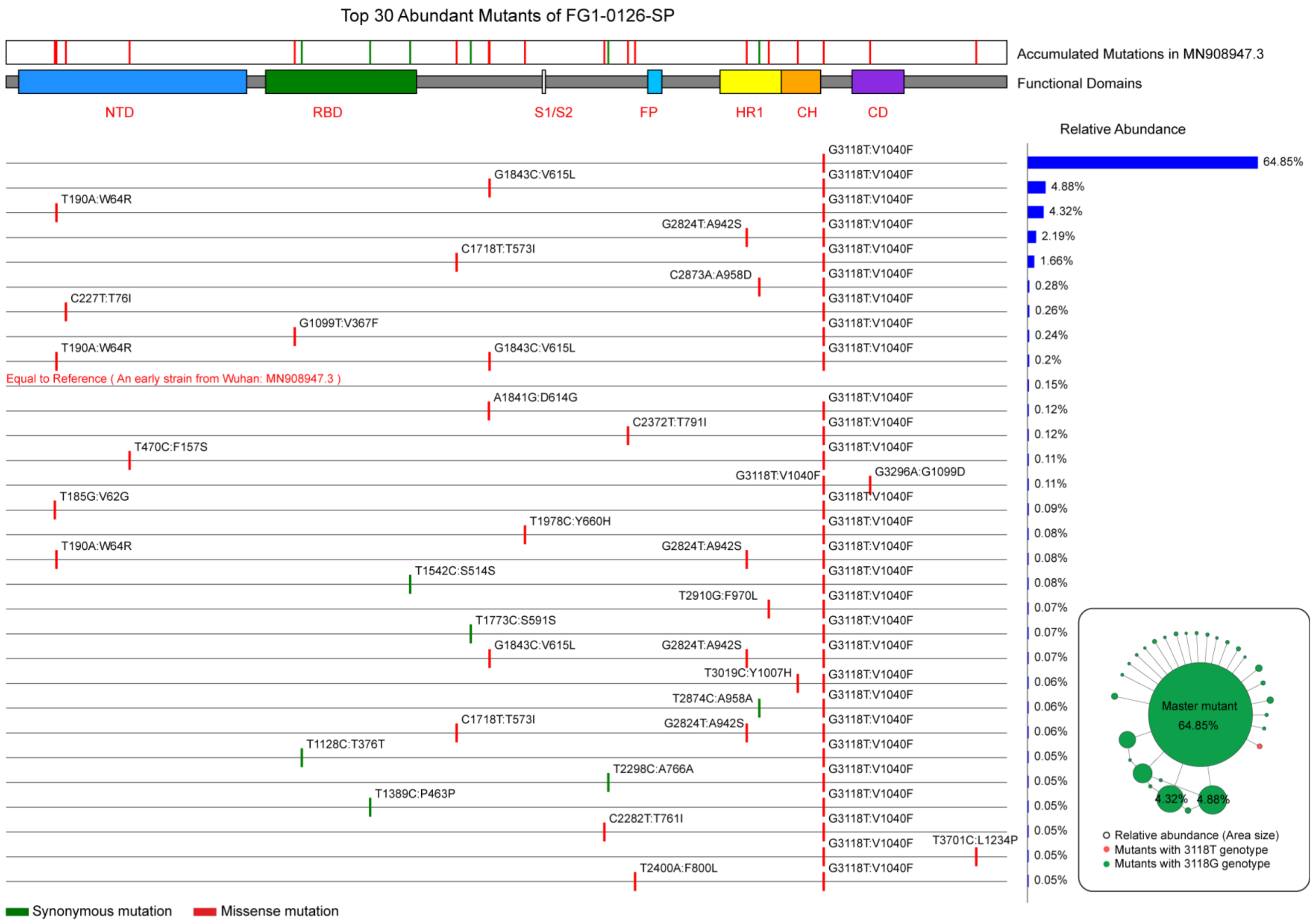
Top 30 Abundant Mutants of FG1-0126-SP
Accumulated Mutations in MN908947.3
Functional Domains
NTD
RBD
S1/S2
FP
HR1
CH
CD
Relative Abundance
G3118T:V1040F
64.85%
G1843C:V615L
G3118T:V1040F
4.88%
T190A:W64R
G3118T:V1040F
4.32%
G2824T:A942S
G3118T:V1040F
2.19%
C1718T:T573I
G3118T:V1040F
1.66%
C2873A:A958D
G3118T:V1040F
0.28%
C227T:T76I
G3118T:V1040F
0.26%
G1099T:V367F
G3118T:V1040F
0.24%
T190A:W64R
G1843C:V615L
G3118T:V1040F
0.2%
Equal to Reference (An early strain from Wuhan: MN908947.3)
0.15%
A1841G:D614G
G3118T:V1040F
0.12%
C2372T:T791I
G3118T:V1040F
0.12%
T470C:F157S
G3118T:V1040F
0.11%
G3118T:V1040F
G3296A:G1099D
0.11%
T185G:V62G
G3118T:V1040F
0.09%
T1978C:Y660H
G3118T:V1040F
0.08%
T190A:W64R
G2824T:A942S
G3118T:V1040F
0.08%
T1542C:S514S
G3118T:V1040F
0.08%
T2910G:F970L
G3118T:V1040F
0.07%
T1773C:S591S
G3118T:V1040F
0.07%
G1843C:V615L
G2824T:A942S
G3118T:V1040F
0.07%
T3019C:Y1007H
G3118T:V1040F
0.06%
T2874C:A958A
G3118T:V1040F
0.06%
C1718T:T573I
G2824T:A942S
G3118T:V1040F
0.06%
T1128C:T376T
G3118T:V1040F
0.05%
T2298C:A766A
G3118T:V1040F
0.05%
T1389C:P463P
G3118T:V1040F
0.05%
C2282T:T761I
G3118T:V1040F
0.05%
G3118T:V1040F
T3701C:L1234P
0.05%
T2400A:F800L
G3118T:V1040F
0.05%
Master mutant
64.85%
4.32%
4.88%
Relative abundance (Area size)
Mutants with 3118T genotype
Mutants with 3118G genotype
Synonymous mutation
Missense mutation

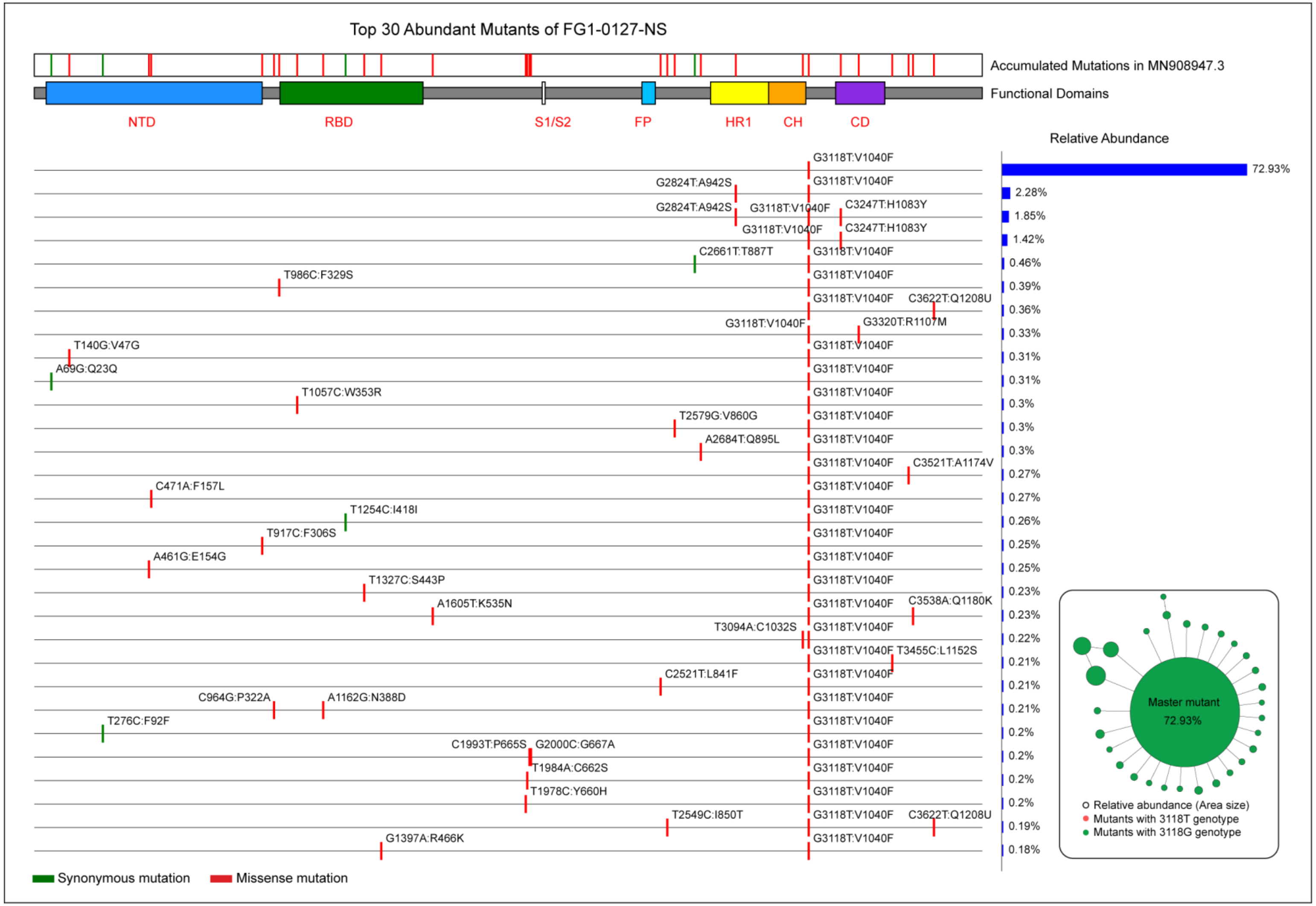

Top 30 Abundant Mutants of FG1-0127-NS
Accumulated Mutations in MN908947.3
Functional Domains
NTD
RBD
S1/S2
FP
HR1
CH
CD
Relative Abundance
Synonymous mutation
Missense mutation
Master mutant
72.93%
Relative abundance (Area size)
Mutants with 3118T genotype
Mutants with 3118G genotype

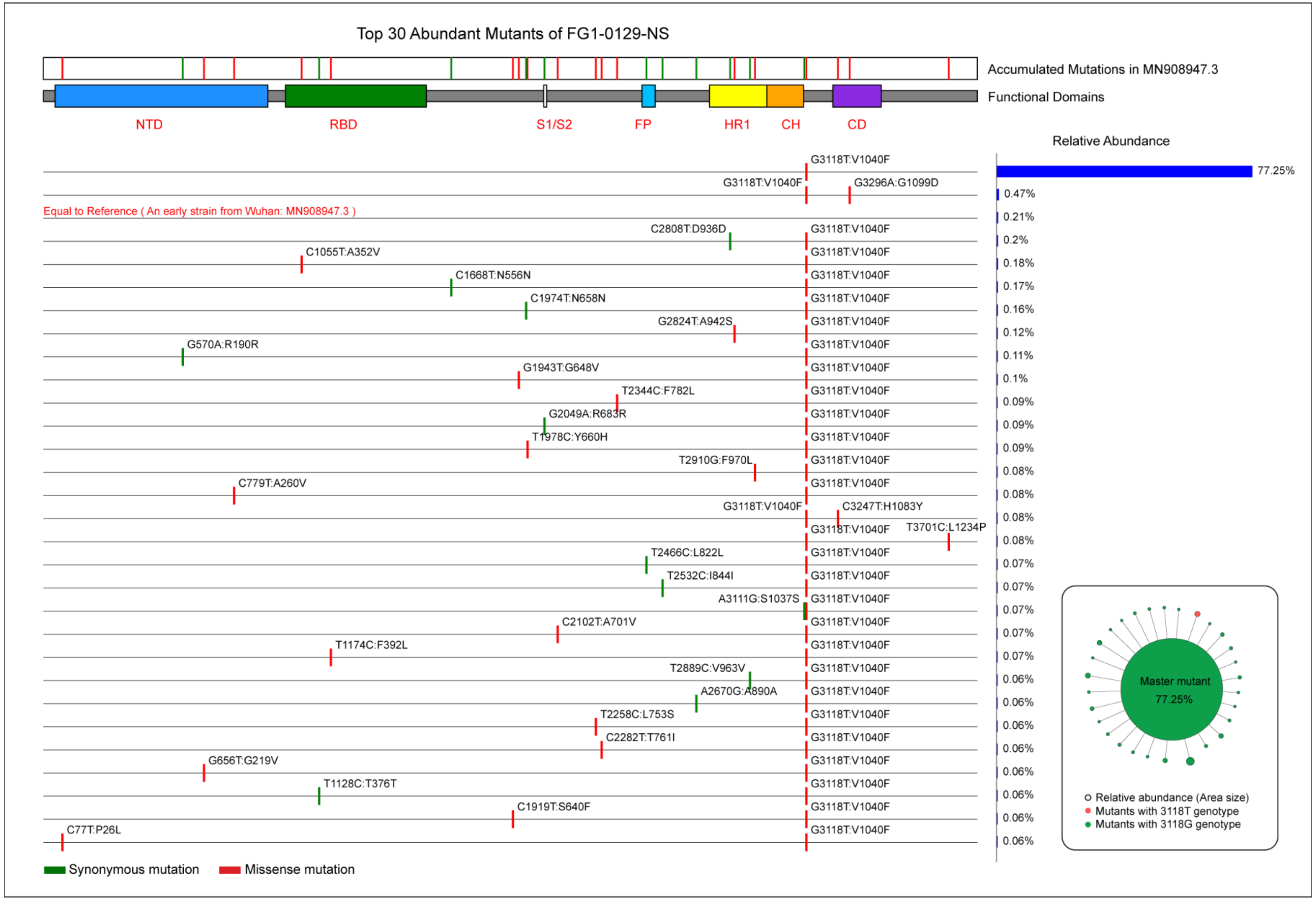
Top 30 Abundant Mutants of FG1-0129-NS
Accumulated Mutations in MN908947.3
Functional Domains
NTD
RBD
S1/S2
FP
HR1
CH
CD
Relative Abundance
G3118T:V1040F
77.25%
G3118T:V1040F
G3296A:G1099D
0.47%
Equal to Reference (An early strain from Wuhan: MN908947.3)
0.21%
C2808T:D936D
0.2%
C1055T:A352V
0.18%
C1668T:N556N
0.17%
C1974T:N658N
0.16%
G2824T:A942S
0.12%
G570A:R190R
0.11%
G1943T:G648V
0.1%
T2344C:F782L
0.09%
G2049A:R683R
0.09%
T1978C:Y660H
0.09%
T2910G:F970L
0.08%
C779T:A260V
0.08%
C3247T:H1083Y
0.08%
T3701C:L1234P
0.08%
T2466C:L822L
0.07%
T2532C:I844I
0.07%
A3111G:S1037S
0.07%
C2102T:A701V
0.07%
T1174C:F392L
0.07%
T2889C:V963V
0.06%
A2670G:A890A
0.06%
T2258C:L753S
0.06%
C2282T:T761I
0.06%
G656T:G219V
0.06%
T1128C:T376T
0.06%
C1919T:S640F
0.06%
C77T:P26L
0.06%
Master mutant
77.25%
Relative abundance (Area size)
Mutants with 3118T genotype
Mutants with 3118G genotype
Synonymous mutation
Missense mutation

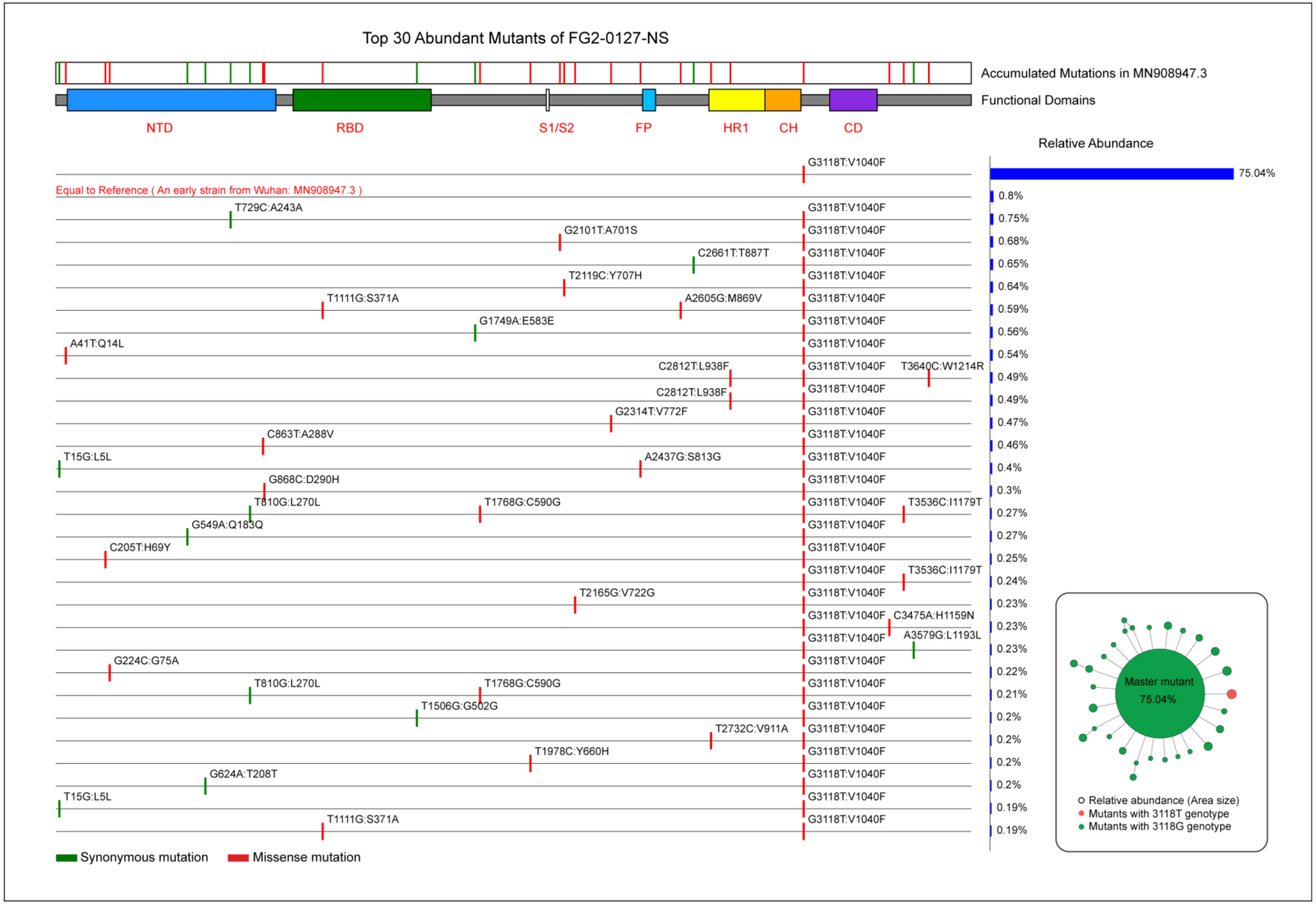
Top 30 Abundant Mutants of FG2-0127-NS
Accumulated Mutations in MN908947.3
Functional Domains
NTD
RBD
S1/S2
FP
HR1
CH
CD
Relative Abundance
G3118T:V1040F
75.04%
Equal to Reference (An early strain from Wuhan: MN908947.3)
0.8%
T729C:A243A
G3118T:V1040F
0.75%
G2101T:A701S
G3118T:V1040F
0.68%
C2661T:T887T
G3118T:V1040F
0.65%
T2119C:Y707H
G3118T:V1040F
0.64%
T1111G:S371A
A2605G:M869V
G3118T:V1040F
0.59%
G1749A:E583E
G3118T:V1040F
0.56%
A41T:Q14L
G3118T:V1040F
0.54%
C2812T:L938F
G3118T:V1040F
T3640C:W1214R
0.49%
C2812T:L938F
G3118T:V1040F
0.49%
G2314T:V772F
G3118T:V1040F
0.47%
C863T:A288V
G3118T:V1040F
0.46%
T15G:L5L
A2437G:S813G
G3118T:V1040F
0.4%
G868C:D290H
G3118T:V1040F
0.3%
T810G:L270L
T1768G:C590G
G3118T:V1040F
T3536C:I1179T
0.27%
G549A:Q183Q
G3118T:V1040F
0.27%
C205T:H69Y
G3118T:V1040F
0.25%
G3118T:V1040F
T3536C:I1179T
0.24%
T2165G:V722G
G3118T:V1040F
0.23%
G3118T:V1040F
C3475A:H1159N
0.23%
G3118T:V1040F
A3579G:L1193L
0.23%
G224C:G75A
G3118T:V1040F
0.22%
T810G:L270L
T1768G:C590G
G3118T:V1040F
0.21%
T1506G:G502G
G3118T:V1040F
0.2%
T2732C:V911A
G3118T:V1040F
0.2%
T1978C:Y660H
G3118T:V1040F
0.2%
G624A:T208T
G3118T:V1040F
0.2%
T15G:L5L
G3118T:V1040F
0.19%
T1111G:S371A
G3118T:V1040F
0.19%
Master mutant
75.04%
Relative abundance (Area size)
Mutants with 3118T genotype
Mutants with 3118G genotype
Synonymous mutation
Missense mutation

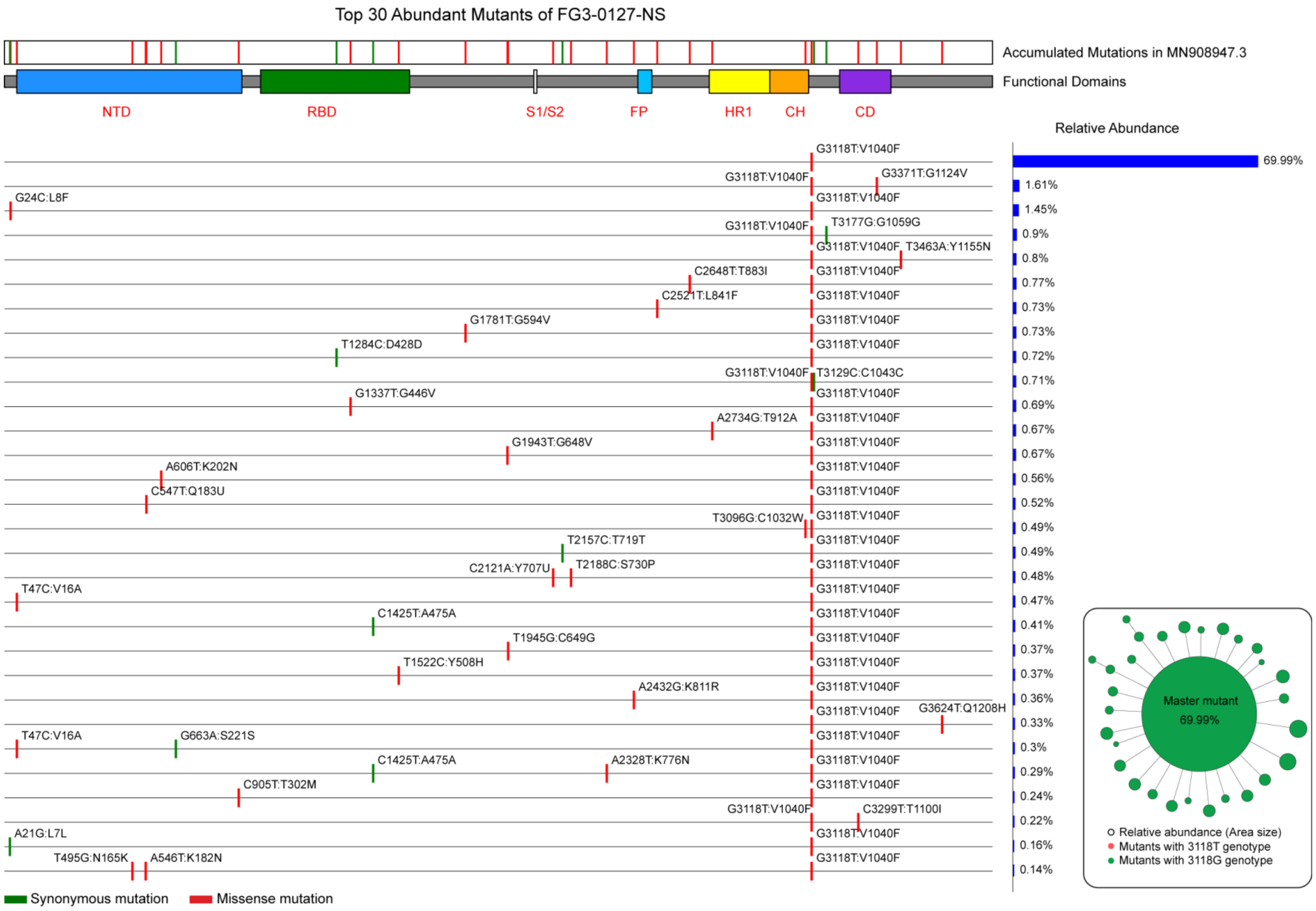
Top 30 Abundant Mutants of FG3-0127-NS
Accumulated Mutations in MN908947.3
Functional Domains
NTD
RBD
S1/S2
FP
HR1
CH
CD
Relative Abundance
G3118T:V1040F
69.99%
G3371T:G1124V
1.61%
G24C:L8F
1.45%
T3177G:G1059G
0.9%
T3463A:Y1155N
0.8%
C2648T:T883I
0.77%
C2521T:L841F
0.73%
G1781T:G594V
0.73%
T1284C:D428D
0.72%
T3129C:C1043C
0.71%
G1337T:G446V
0.69%
A2734G:T912A
0.67%
G1943T:G648V
0.67%
A606T:K202N
0.56%
C547T:Q183U
0.52%
T3096G:C1032W
0.49%
T2157C:T719T
0.49%
C2121A:Y707U
T2188C:S730P
0.48%
T47C:V16A
0.47%
C1425T:A475A
0.41%
T1945G:C649G
0.37%
T1522C:Y508H
0.37%
A2432G:K811R
0.36%
G3624T:Q1208H
0.33%
T47C:V16A
G663A:S221S
0.3%
C1425T:A475A
A2328T:K776N
0.29%
C905T:T302M
0.24%
C3299T:T1100I
0.22%
A21G:L7L
0.16%
T495G:N165K
A546T:K182N
0.14%
Master mutant
69.99%
Relative abundance (Area size)
Mutants with 3118T genotype
Mutants with 3118G genotype
Synonymous mutation
Missense mutation

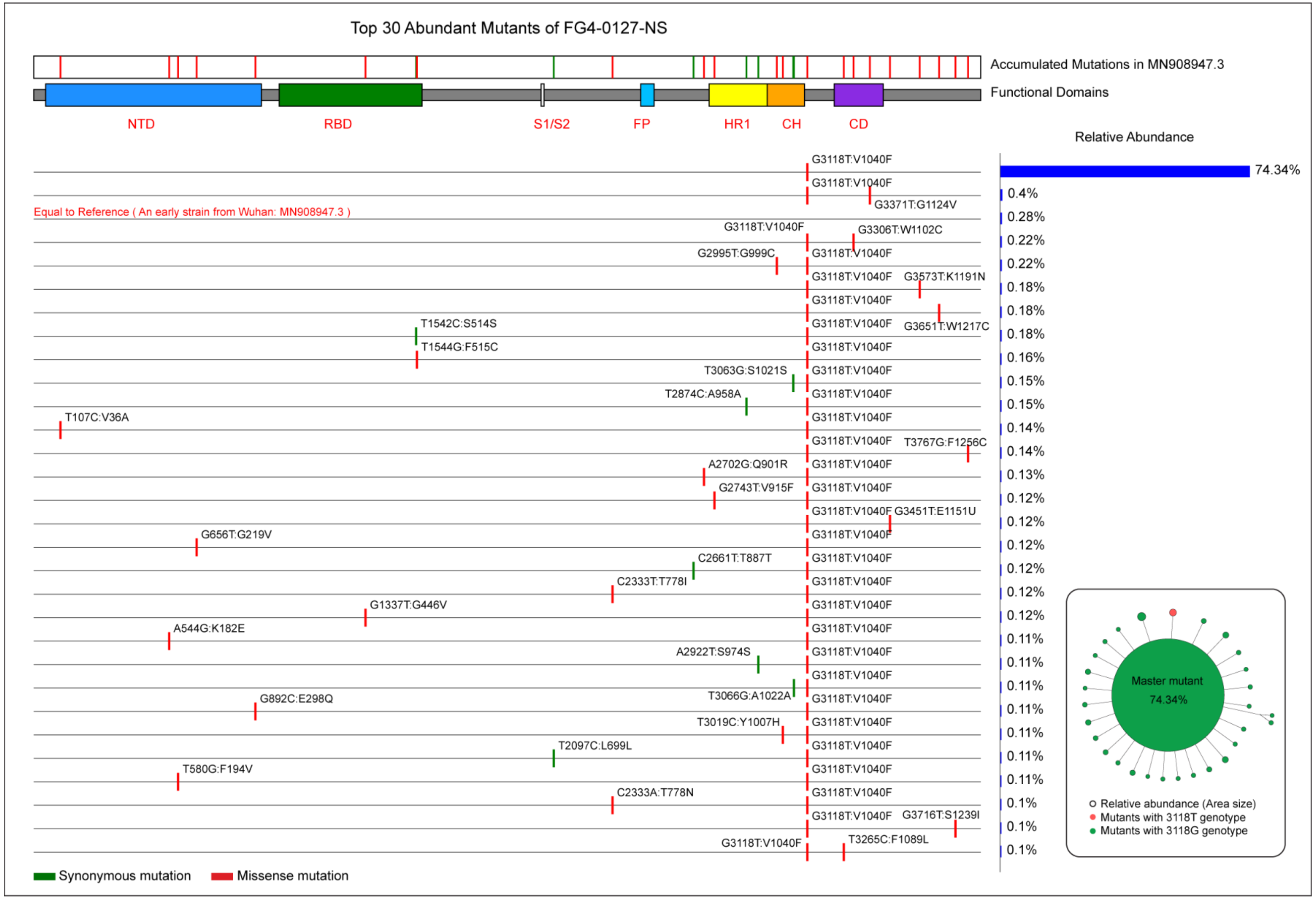
Top 30 Abundant Mutants of FG4-0127-NS
Accumulated Mutations in MN908947.3
Functional Domains
NTD
RBD
S1/S2
FP
HR1
CH
CD
Relative Abundance
Equal to Reference (An early strain from Wuhan: MN908947.3)
G3118T:V1040F
G3371T:G1124V
G3306T:W1102C
G2995T:G999C
G3573T:K1191N
T1542C:S514S
T1544G:F515C
G3651T:W1217C
T3063G:S1021S
T2874C:A958A
T107C:V36A
T3767G:F1256C
A2702G:Q901R
G2743T:V915F
G3451T:E1151U
G656T:G219V
C2661T:T887T
C2333T:T778I
G1337T:G446V
A544G:K182E
A2922T:S974S
T3066G:A1022A
G892C:E298Q
T3019C:Y1007H
T2097C:L699L
T580G:F194V
C2333A:T778N
G3716T:S1239I
T3265C:F1089L
74.34%
0.4%
0.28%
0.22%
0.22%
0.18%
0.18%
0.18%
0.16%
0.15%
0.15%
0.14%
0.14%
0.13%
0.12%
0.12%
0.12%
0.12%
0.12%
0.12%
0.11%
0.11%
0.11%
0.11%
0.11%
0.11%
0.11%
0.1%
0.1%
0.1%
Master mutant
74.34%
Relative abundance (Area size)
Mutants with 3118T genotype
Mutants with 3118G genotype
Synonymous mutation
Missense mutation

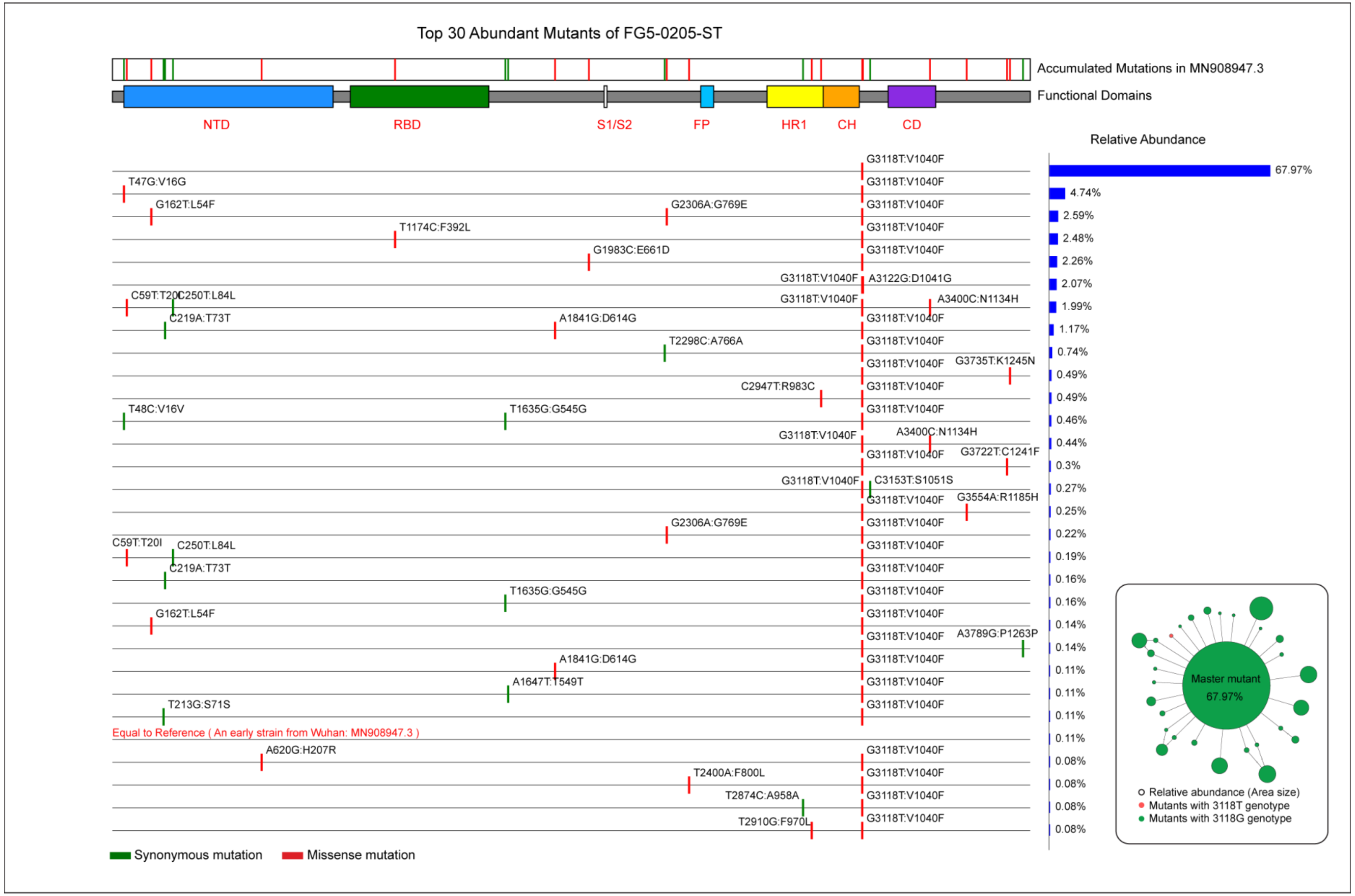
Top 30 Abundant Mutants of FG5-0205-ST
Accumulated Mutations in MN908947.3
Functional Domains
NTD
RBD
S1/S2
FP
HR1
CH
CD
Relative Abundance
G3118T:V1040F
T47G:V16G
G162T:L54F
G2306A:G769E
T1174C:F392L
G1983C:E661D
A3122G:D1041G
C59T:T20I
C250T:L84L
A3400C:N1134H
C219A:T73T
A1841G:D614G
T2298C:A766A
G3735T:K1245N
C2947T:R983C
T48C:V16V
T1635G:G545G
G3722T:C1241F
C3153T:S1051S
G3554A:R1185H
A3789G:P1263P
A1647T:T549T
T213G:S71S
Equal to Reference (An early strain from Wuhan: MN908947.3)
A620G:H207R
T2400A:F800L
T2874C:A958A
T2910G:F970L
67.97%
4.74%
2.59%
2.48%
2.26%
2.07%
1.99%
1.17%
0.74%
0.49%
0.49%
0.46%
0.44%
0.3%
0.27%
0.25%
0.22%
0.19%
0.16%
0.16%
0.14%
0.14%
0.11%
0.11%
0.11%
0.11%
0.08%
0.08%
0.08%
0.08%
Master mutant
67.97%
Relative abundance (Area size)
Mutants with 3118T genotype
Mutants with 3118G genotype
Synonymous mutation
Missense mutation

Figure S2 Details of the top 30 abundant mutants of each sample. Each line represented a kind of mutant, green lines were the missense mutations and the red colors were synonymous mutations. The top rectangle represents the cumulative variations from quasispecies mutants. Functional domain of the spike gene were marked with different colors: NTD, N-terminal domain; RBD, receptor-binding domain; S1/S2, protease cleavage site; FP, fusion peptide; HR1, heptad repeat 1; CH, central helix; CD, connector domain; HR2, heptad repeat 2; TM, transmembrane domain; CT, cytoplasmic tail. The rightmost bar plots showed the proportion of mutants (abundance). Results of haplotype clustering were listed at the right bottom, where mutation spectrums centered by the master one were identified.

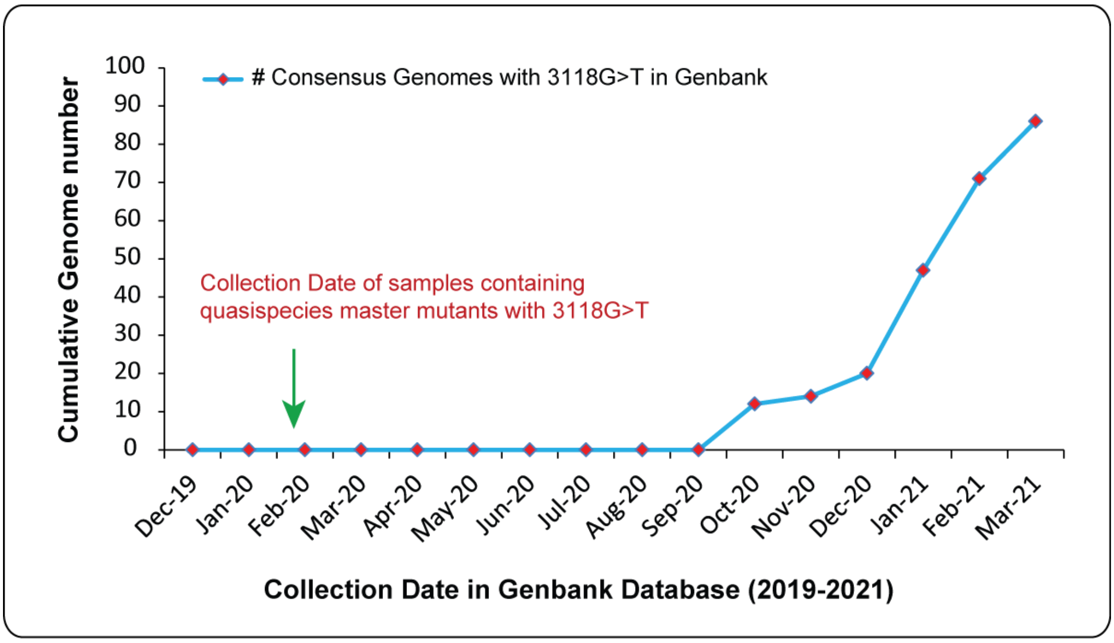

Figure S3 The occurrence statistics of the consensus genomes with 3118G>T in Genbank database (by March 2021). The results showed that 3118G>T was never emerged before October 2020 that was 10 month after the sampling date of the current study.

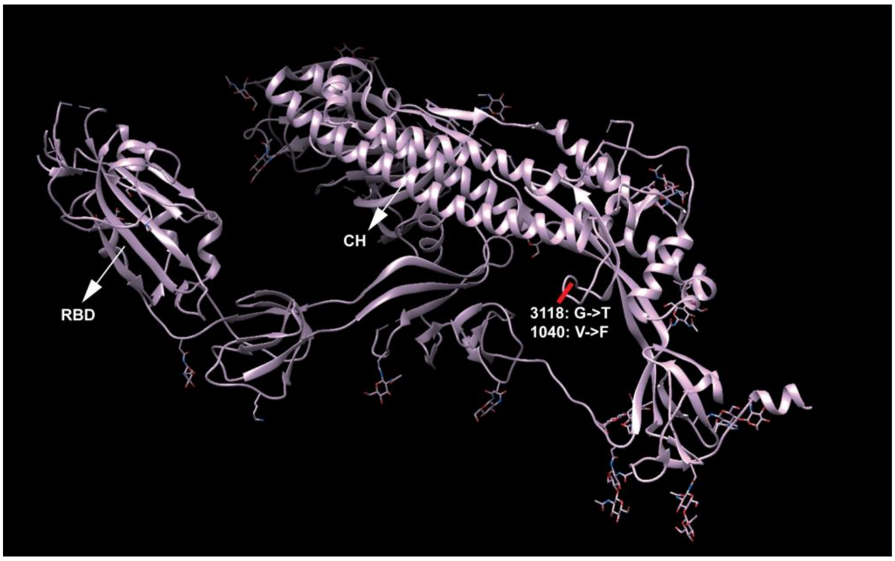

Figure S4 The position of 3118G>T on the spike protein (3D ribbon structure). The corresponding amino acid is highlighted by a red line and some potential functional domains are marked with arrow. The nucleotide of 3118T was located in the turn site between CH and CD sub-domains.

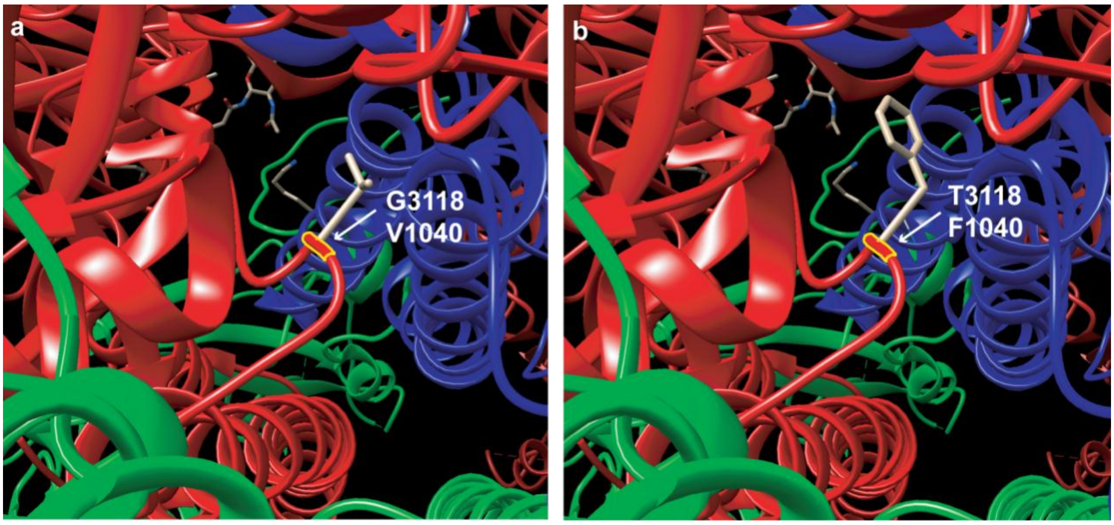

Figure S5 Simulation for the amino acid changes of the 3118 G>T. Part **a** showed the nucleotide of SG master mutant G (same with the early ancestor strain), and part **b** showed the nucleotide of FG master mutant T. The location of variation site was highlighted by yellow color, the changed angle of R-group was simulated by Chimera with the Posterior probability=0.30.

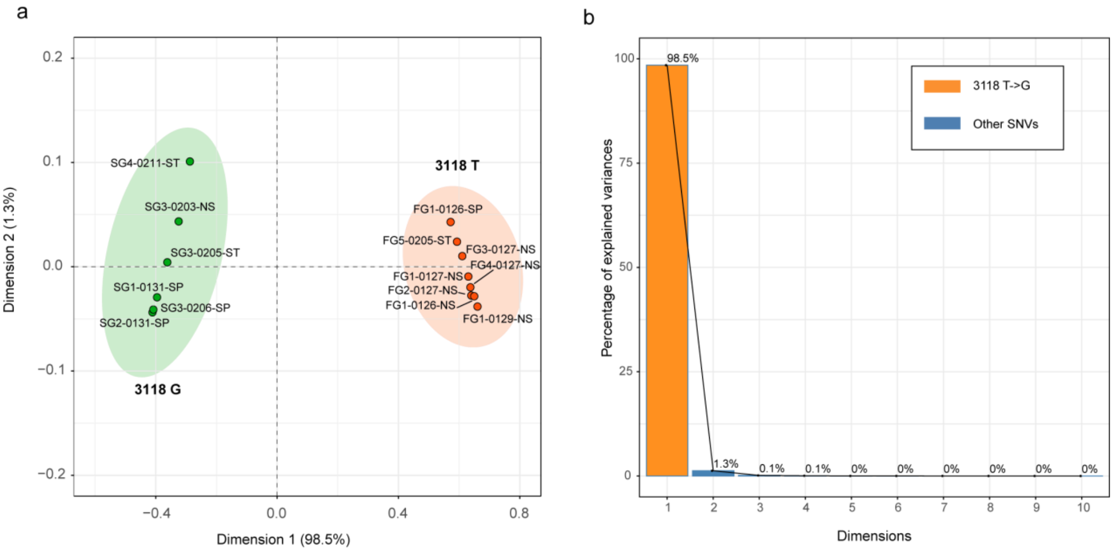

Figure S6 Principal component analysis for all samples. (**a**) Two clusters were identified based on all SNVs of the top 30 abundant mutants. (**b**) The SNV 3118T>G as the first dimension could fully distinguish the two single-source infection groups and made more than 98% contributions to the grouping.

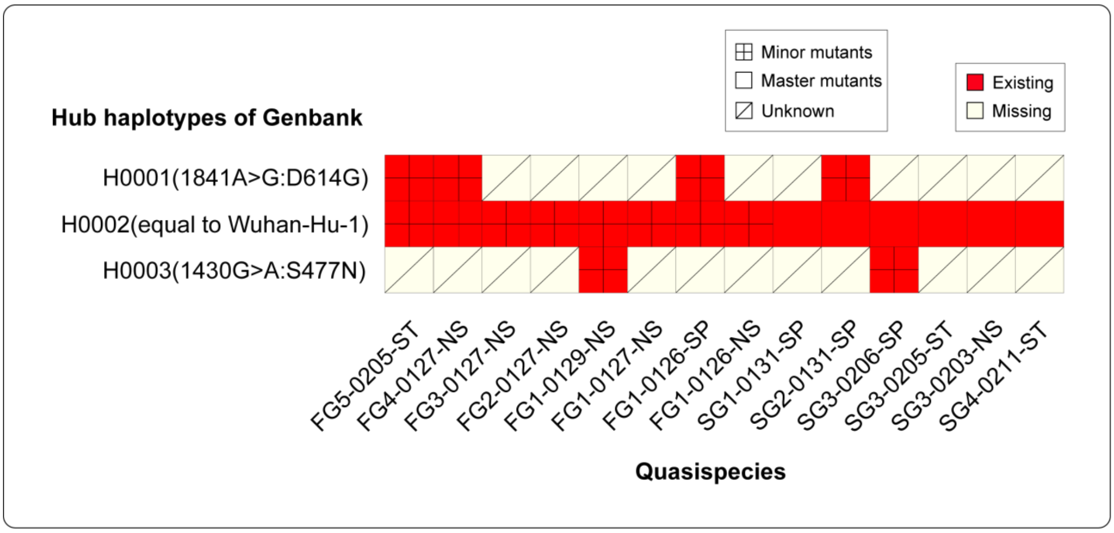

Figure S7 Occurrence of three hub spike haplotypes of Genbank in the quasispecies mutants. Three haplotypes reflected for three master mutants within patients, and all these master mutants had already existed in the minor mutants of early infected patients.

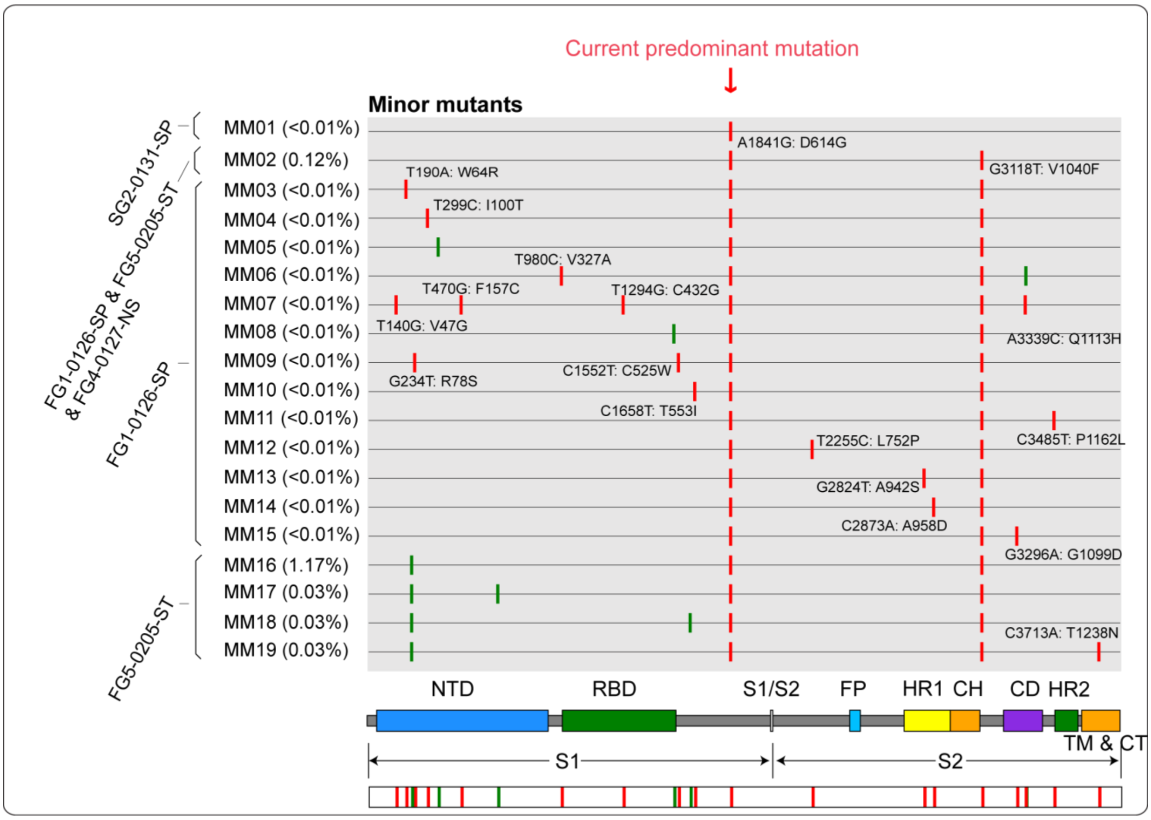

Figure S8 Detecting a currently predominant SNV 1841A>G (D614G) in the minor mutants of the quasispecies. The nucleotide 1841G (amino acid G614) had already existed in 19 minor mutants from 4 early infected patients.

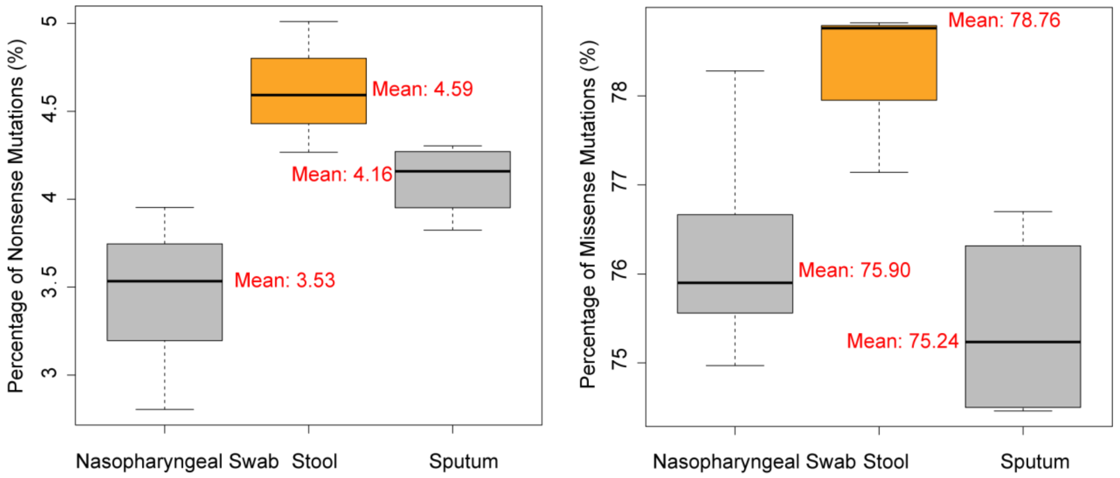

Figure S9 Box plots of the nonsense and missense mutation rate for three sampling sites. Both percentage of nonsense and missense mutation showed obviously higher in samples of stools. The mean values were marked on the figure.

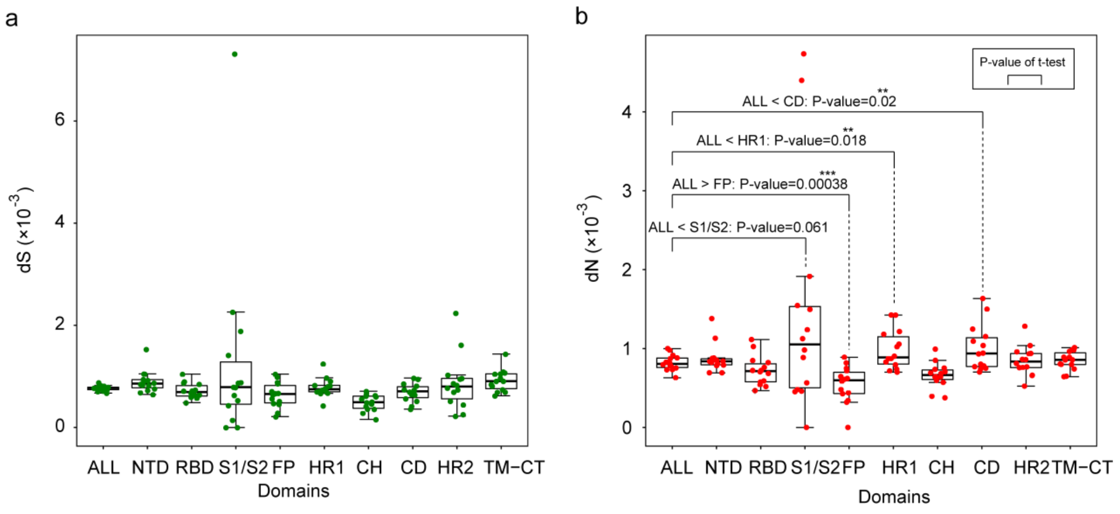

Figure S10 Box plots of the dN and dS value. The mean dS values of each domain are almost the same (**a**), while the mean dN values of three domains of FP, HR1, and CD were significantly larger than the whole gene level (t-test) (**b**).
